# Spectrum of Ocular Manifestations in CLN2-Associated Batten (Jansky-Bielschowsky) Disease Correlate with Advancing Age and Deteriorating Neurological Function

**DOI:** 10.1371/journal.pone.0073128

**Published:** 2013-08-28

**Authors:** Anton Orlin, Dolan Sondhi, Matthew T. Witmer, Matthew M. Wessel, Jason G. Mezey, Stephen M. Kaminsky, Neil R. Hackett, Kaleb Yohay, Barry Kosofsky, Mark M. Souweidane, Michael G. Kaplitt, Donald J. D’Amico, Ronald G. Crystal, Szilárd Kiss

**Affiliations:** 1 Department of Ophthalmology, Weill Cornell Medical College, New York, New York, United States of America; 2 Department of Genetic Medicine, Weill Cornell Medical College, New York, New York, United States of America; 3 Department of Biological Statistics and Computational Biology, Cornell University, Ithaca, New York, United States of America; 4 Department of Neurology, Weill Cornell Medical College, New York, New York, United States of America; Faculty of Medicine University of Leipzig, Germany

## Abstract

**Background:**

Late infantile neuronal ceroid lipofuscinosis (LINCL), one form of Batten’s disease is a progressive neurodegenerative disorder resulting from a *CLN2* gene mutation. The spectrum of ophthalmic manifestations of LINCL and the relationship with neurological function has not been previously described.

**Methods:**

Patients underwent ophthalmic evaluations, including anterior segment and dilated exams, optical coherence tomography, fluorescein and indocyanine green angiography. Patients were also assessed with the LINCL Neurological Severity Scale. Ophthalmic findings were categorized into one of five severity scores, and the association of the extent of ocular disease with neurological function was assessed.

**Results:**

Fifty eyes of 25 patients were included. The mean age at the time of exam was 4.9 years (range 2.5 to 8.1). The mean ophthalmic severity score was 2.6 (range 1 to 5). The mean neurological severity score was 6.1 (range 2 to 11). Significantly more severe ophthalmic manifestations were observed among older patients (p<0.005) and patients with more severe neurological findings (p<0.03). A direct correlation was found between the Ophthalmic Severity Scale and the Weill Cornell Neurological Scale (p<0.002). A direct association was also found between age and the ophthalmic manifestations (p<0.0002), with older children having more severe ophthalmic manifestations.

**Conclusions:**

Ophthalmic manifestations of LINCL correlate closely with the degree of neurological function and the age of the patient. The newly established LINCL Ophthalmic Scale may serve as an objective marker of LINCL severity and disease progression, and may be valuable in the evaluation of novel therapeutic strategies for LINCL, including gene therapy.

## Introduction

Neuronal ceroid lipofuscinoses (NCL), collectively the most common progressive neurodegenerative disorders of childhood, comprise a heterogeneous group of lysosomal storage diseases [1–4]. These autosomal recessive syndromes result from mutations in at least 11 genes, encoding for either soluble lysosomal enzymes or transmembrane proteins (*CLN1* through *CLN10*, and *CLCN7*) [4,5]. NCLs are grouped into congenital, infantile, late infantile (LINCL), juvenile (JNCL) and adult onset forms, depending on the specific gene mutations [1,4]. They are all characterized by an accumulation of hydrophobic autofluorescent material in the cytoplasm of neurons [4,6,7].

Mutations in the *CLN2* gene, on chromosome 11p15, lead to classic LINCL, also referred to as Batten disease or Jansky–Bielschowsky disease [8–10]. *CLN2* encodes the soluble CLN2 protein tripeptidyl peptidase 1 (TPP1), a lysosomal hydrolase that removes tripeptides from the N-terminus of small polypeptides [8,11,12]. *CLN2* mutations result in an accumulation of curvilinear profiles in lysosomal residual bodies, the hallmark of LINCL [8]. The relationship between the accumulation of materials in the lysosomes and neuronal injury has yet to be fully elucidated [4,13].

Clinically, NCLs are characterized by developmental delay, psychomotor regression, ataxia, seizures, visual decline and premature death. The early clinical course of LINCL is dominated by seizures and ataxia, onset of which occurs around age 2 to 4. This is followed by cognitive and motor dysfunction, with complete loss of motor function by age 6 and death by mid-childhood [8,14,15]. In contrast to the early manifestations of vision loss in JNCL patients, visual impairment develops late in the disease course of LINCL, when progressive mental regression and gross motor disturbances have already become noticeable [4,8,16].

Vision loss in NCL occurs secondary to a progressive retinal degeneration of uncertain pathophysiology [4,8,16]. Previously reported ophthalmic manifestations, largely in JNCL patients, include a “bull’s eye” maculopathy, retinal pigment epithelial atrophy, fundus autofluorescence abnormalities, peripheral pigmentary disturbances, and a reduced or extinguished electroretinogram [17–25]. The ophthalmic manifestations of LINCL and their relationship with neurological function and the age of the patient have not been previously described.

In this current report, we characterized and categorized the ophthalmic manifestations of 25 LINCL patients with genetically confirmed *CLN2* mutations. Based on these 50 eyes, we developed the Weill Cornell LINCL Ophthalmic Severity Scale and correlated the ophthalmic findings with the previously validated Weill Cornell LINCL Clinical Neurological Scale [15] and the age of the patient.

## Methods

### Ethics Statement

Twenty-five patients with genetically confirmed LINCL underwent comprehensive ophthalmic examinations under anesthesia at Weill Cornell Medical College and New, York/Presbyterian Hospital. The ophthalmic evaluations were part of a thorough screening program of LINCL patients for inclusion in an on-going prospective NIH sponsored clinical trial of brain directed gene therapy for the treatment of neurological manifestations of LINCL (www.clinicaltrials.gov). Subjects were recruited and written informed consent obtained under protocols approved by The Weill Cornell Medical College and New, York/Presbyterian Hospital Institutional Review Boards (IRB) according to local and national IRB guidelines.

### Ophthalmologic Parameters

Given the severity of motor and cognitive abnormalities associated with LINCL, all patients were examined while under sedation. The ophthalmic evaluation included anterior segment and dilated exam, fundus photography (RETCAM), spectral domain optical coherence tomography (OCT, Heidelberg Engineering), fluorescein angiography (FA, Heidelberg Engineering and RETCAM) and indocyanine green angiography (ICGA, Heidelberg Engineering).

The ocular exam, OCT, FA, and ICGA were used to establish the extent of retinal degeneration in each patient. Based on the evaluation of both eyes, each patient was given one ophthalmic score. The ophthalmic manifestations were divided into one of five severity categories – an ophthalmic score of 1, representing a normal fundus with no clinically discernable evidence of retinal degeneration, and an ophthalmic score of 5, exhibiting the most severe, widespread retinal degeneration. Using the collected images (fundus photograph, OCT, FA, and ICGA), four trained, independent, masked retina specialists graded the severity of the retinal degeneration in each of the 50 LINCL eyes (which were presented to the graders in random fashion.) The mean of the 4 expert evaluators was used to determine the ophthalmic severity grade for each eye of each patient. Based on the ophthalmic severity grade of each eye, the patient was assigned an ophthalmic severity score, which was ultimately used for the statistical analysis for correlation with clinical and neurological parameters.

### Clinical Neurologic Parameters

In addition to the ophthalmic evaluations, neurological deterioration was assessed using the previously described Weill Cornell LINCL neurological scale [15]. The index of disability on the neurological score ranges from a maximum score of 12 for normal neurological function, and a minimum of 0 for most severe neurological debility. To determine the neurological function score, each patient was assessed on the LINCL scale by a pediatric neurologist. The assessment was videotaped and given a randomized number. The video was then scored by 3 masked, independent, trained, pediatric neurologists and then averaged, to obtain the final score.

### Statistical Analysis

The correlation among ophthalmic graders and the association of the extent of ophthalmic severity with neurological deterioration and the age of the patient was assessed using a non-parametric approach. Kendall’s rank correlation was calculated for all pairwise comparisons among graders, as well as between ophthalmic severity-neurological deterioration and ophthalmic severity-age, and p values were calculated using the "cor. test" function in R for the null hypothesis of no association in each case. We additionally divided the sample into older and younger patients, defined as above and below the median, and for less and more severe neurological deterioration, again using the median, and used a Kruskal-Wallis test in each case to test the null hypothesis of no difference between the groups.

## Results

Fifty eyes of 25 patients were included in the analysis (Table 1). All 25 patients had genetically confirmed *CLN2* mutations and carried the diagnosis of LINCL. All 50 eyes underwent a comprehensive ocular exam along with FA and color fundus photography (Figure S1). Thirty-four of 50 eyes (68%) had an OCT, while 30 of the 50 eyes (60%) had an ICGA.

**Table 1:** Characteristics of Patients with Late Infantile Neuronal Ceroid Lipofuscinosis (LINCL) Undergoing Ophthalmic Evaluations^1^.

**Patient**	**Age at exam (in months)**	**Genotype**	**Neurologic score**	**Ophthalmic evaluation^2^**	**Ophthalmic score^3^**
1	55	C3670T, G3556C	5.5±0.6	Color, FA	1.0±0.0
2	50	C3670T, G3556C	9.0±0.0	Color, FA	1.0±0.0
3	83	G3556C, G3556C	7.5±0.6	Color, FA, OCT	5.0±0.0
4	63	C3670T, G3556C	3.0±0.0	Color, FA, ICGA, OCT	5.0±0.0
5	55	C3670T, G4655A	6.0±0.0	Color, FA	3.8±0.5
6	57	C3670T, G3556C	5.0±0.0	Color, FA, OCT	4.0±0.0
7	59	G3556C, Other	7.0±1.0	Color, FA, ICGA, OCT	3.0±0.0
8	63	C3670T, G3556C	3.0±0.0	Color, FA, ICGA, OCT	4.0±0.0
9	65	G3556C, Other	6.0±1.2	Color, FA, ICGA, OCT	4.0±0.0
10	63	G3556C, Other	7.3±0.6	Color, FA, ICGA, OCT	2.0±0.0
11	39	C3670T, G3556C	9.7±0.6	Color, FA, ICGA, OCT	1.0±0.0
12	52	G3556C, G4655A	7.0±0.0	Color, FA, ICGA, OCT	1.0±0.0
13	56	G3556C, G3556C	7.7±0.6	Color, FA, ICGA, OCT	1.8±0.0
14	32	G3556C, Other	7.5±0.6	Color, FA, ICGA, OCT	1.0±0.0
15	53	C3670T, C3670T	5.0±0.0	Color, FA, ICGA, OCT	2.0±0.0
16	66	G3556C, Other	4.3±0.5	Color, FA, ICGA, OCT	4.0±0.0
17	48	C3670T, G3556C	5.3±0.5	Color, FA, ICGA, OCT	2.0±0.0
18	63	G3556C, Other	6.7±0.6	Color, FA, ICGA, OCT	2.3±0.5
19	97	G3556C, G3556C	2.0±0.6	Color, FA, ICGA, OCT	5.0±0.0
20	30	G3556C, Other	11.0±0.0	Color, FA, ICGA, OCT	1.0±0.0
21	57	C3670T, C3670T	5.0±0.0	Color, FA	3.5±0.6
22	79	C3670T, Other	4.0±0.0	Color, FA	3.0±0.0
23	58	C3670T, C3670T	7.3±1.0	Color, FA	1.3±0.5
24	58	Other, Other	5.0±0.6	Color, FA	1.5±0.6
25	60	C3670T, Other	5.0±0.5	Color, FA	1.0±0.0

^1^ The age of patient at the time of ophthalmic examination under anesthesia is noted in months. The neurological score was determined using the Weill Cornell LINCL clinical neurological scale as previously described [15]. The higher the neurological score indicated better neurological status. Ophthalmic evaluation of patients included a complete dilated fundus examination, color fundus photography (Color), fluorescein angiogram (FA), indocyanine green angiogram (ICGA), and spectral domain optical coherence tomography (OCT). All 25 patients (50 eyes total) underwent Color and FA. 17 patients (34 eyes, as noted in the Table) also had an OCT. ICGA was performed in 15 patients (30 eyes, as noted in the Table). Ophthalmic score was determined using the Weill Cornell LINCL Ophthalmic Severity Scale (form 1 to 5, least severe to most severe ophthalmic findings) as outlined in Table 2. Figure 1 through 5 illustrate the Standard Images used by the masked graders to determine the severity of ophthalmic findings in each patient.

^2^ Color - Dilated Color Fundus Photography; FA - fluorescein angiogram; ICGA - Indocyanine Green Angiogram; OCT - Spectral Domain Optical Coherence Tomography.

^3^Ophthalmic Score - Average of 4 independent, masked graders.

The mean age of the patients at the time of evaluation was 4.9 yr (median-4.8, mode -5.3, range 2.5 to 8.1). The mean LINCL neurological severity score was 6.1 (median -6.0, mode -5.0, range 2.0 to 11.0).

**Figure 1:**
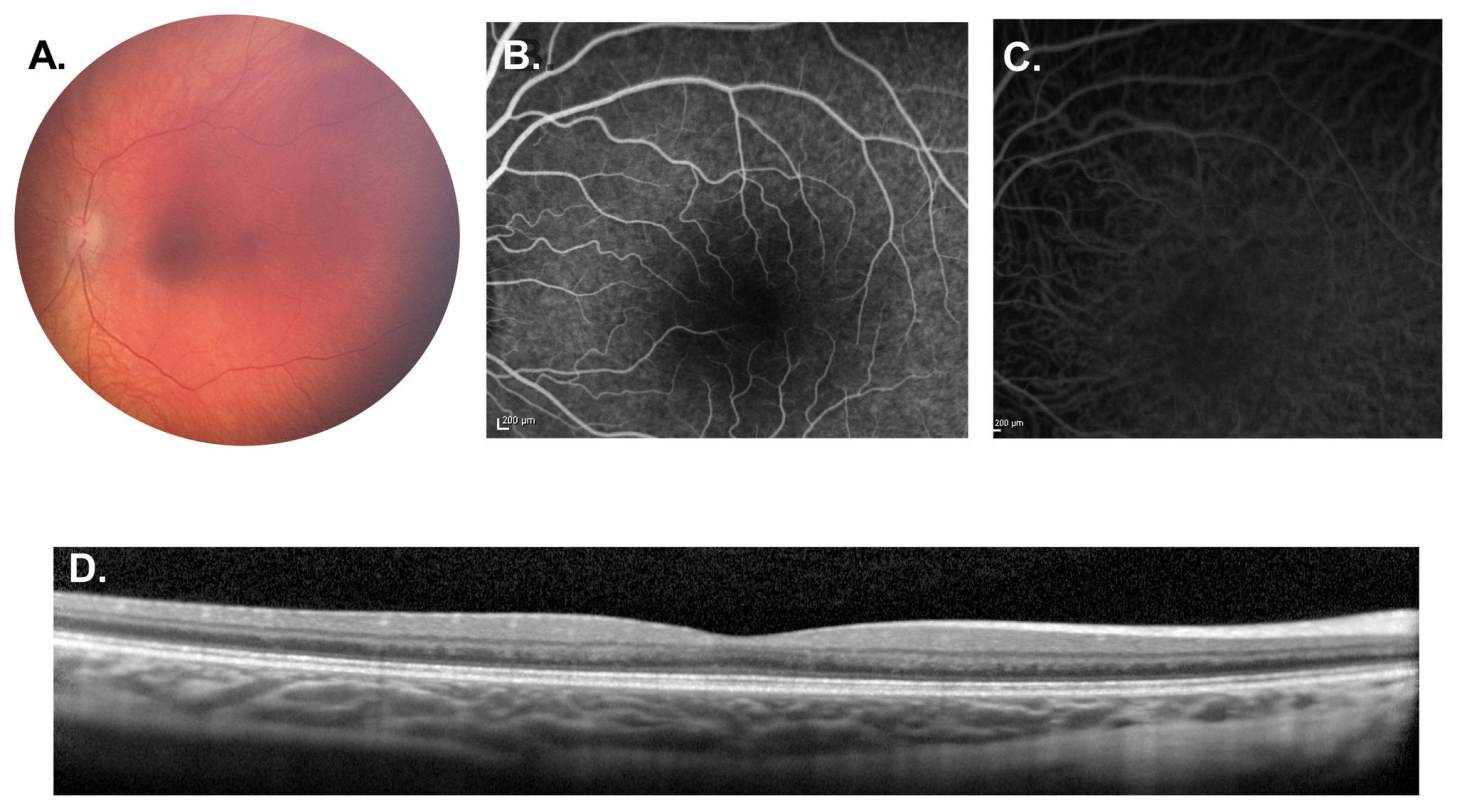
Weill Cornell LINCL Ophthalmic Severity Score 1. **A**. Dilated fundus photograph of patient 14 showing normal appearing optic nerve, vessels and fovea. **B**. Mid-phase FA and **C**. ICGA of patient 20 appear normal. **D**. SD-OCT of the same patient demonstrates normal retinal architecture without disruption of the outer retina. FA – fluorescein angiogram, ICGA - indocyanine green angiogram, SD-OCT – spectral domain optical coherence tomography.

**Figure 2:**
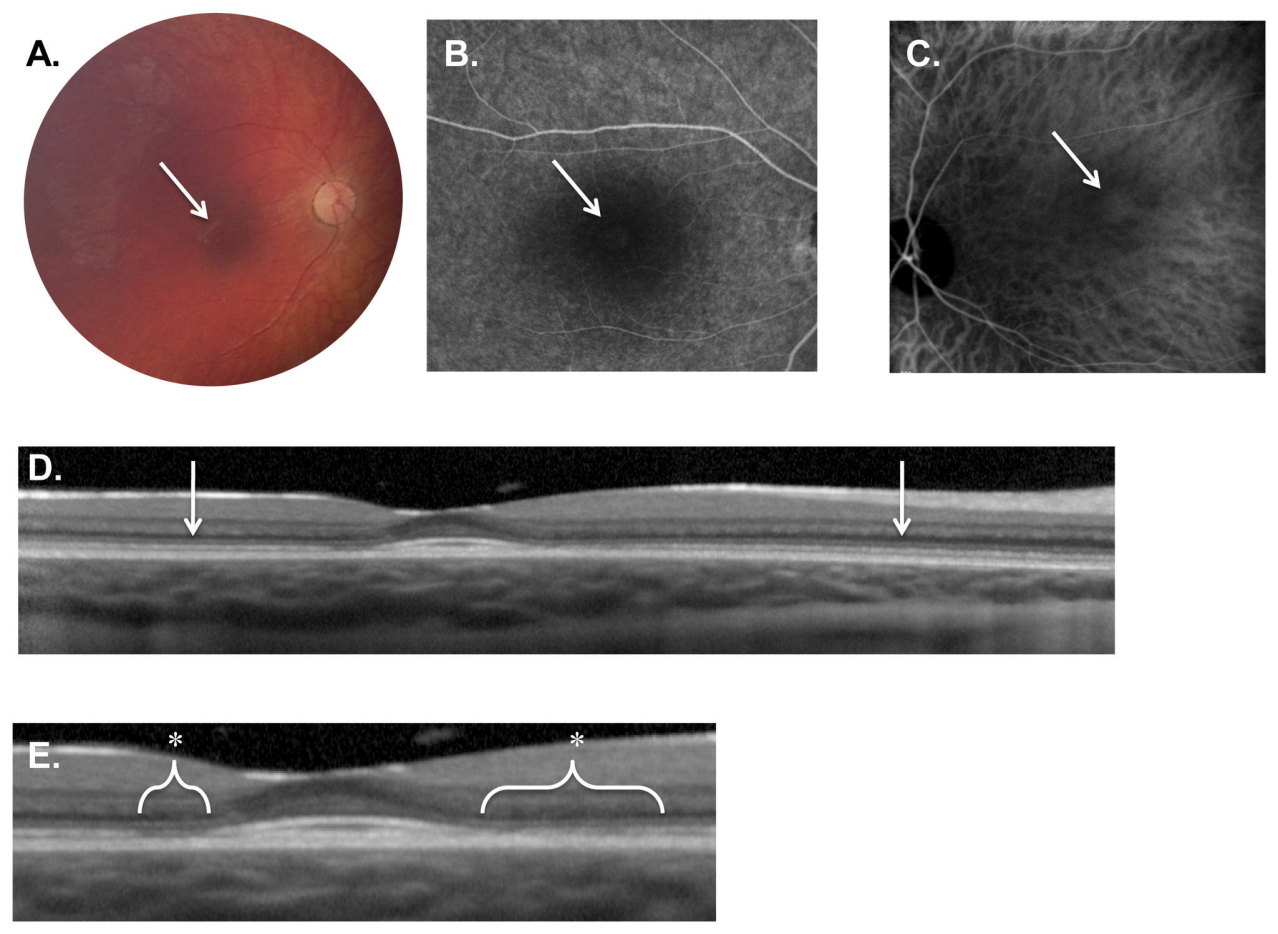
Weill Cornell LINCL Ophthalmic Severity Score 2. **A**. Dilated fundus photograph of Patient 10 reveals subtle pigmentary changes in the fovea (arrow). The optic nerve and vessels appearing normal. **B**. Late-phase FA and C. ICGA of Patient 17 shows a faint area of central hyper-fluorescence (arrow) surrounded by hypo-fluorescence. **D**. SD-OCT of patient 10 demonstrates normal retinal architecture outside the fovea (arrows). **E**. Enlargement of the fovea and para-foveal regions of the same OCT exposes outer retinal abnormalities including the disruption of the ellipsoid hyper-reflective band (*). The external limiting membrane, however, appears intact. FA – fluorescein angiogram, ICGA - indocyanine green angiogram, SD-OCT – spectral domain optical coherence tomography.

**Figure 3:**
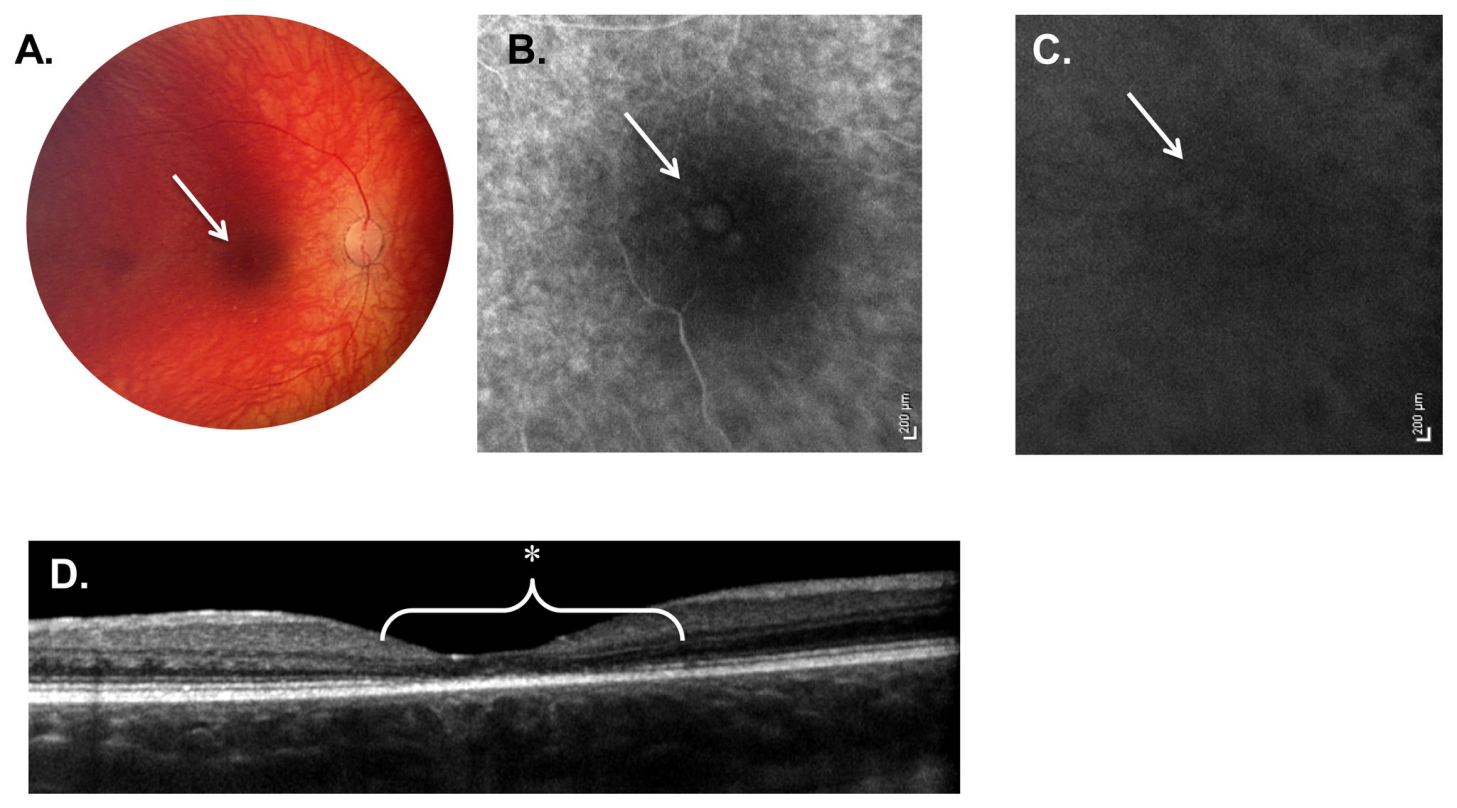
Weill Cornell LINCL Ophthalmic Severity Score 3. **A**. Dilated fundus photograph of patient 7 reveals a central area of pigmentary change in the fovea (arrow). The optic nerve is somewhat pale in appearance with retinal vessels beginning to show signs of attenuation. **B**. Late-phase FA and C. ICGA of the same patient show a more prominent area of central hyper-fluorescence (arrow) surrounded by hypo-fluorescence. A few areas of pinpoint hyper-fluorescence outside this ring are also evident. **D**. SD-OCT of patient 7 demonstrates outer retinal abnormalities, including the disruption of the ellipsoid hyper-reflective band (*) as well as the external limiting membrane. This outer retinal atrophy is confined to the central fovea, extending less than 1 disc diameter. FA – fluorescein angiogram, ICGA - indocyanine green angiogram, SD-OCT – spectral domain optical coherence tomography.

**Figure 4:**
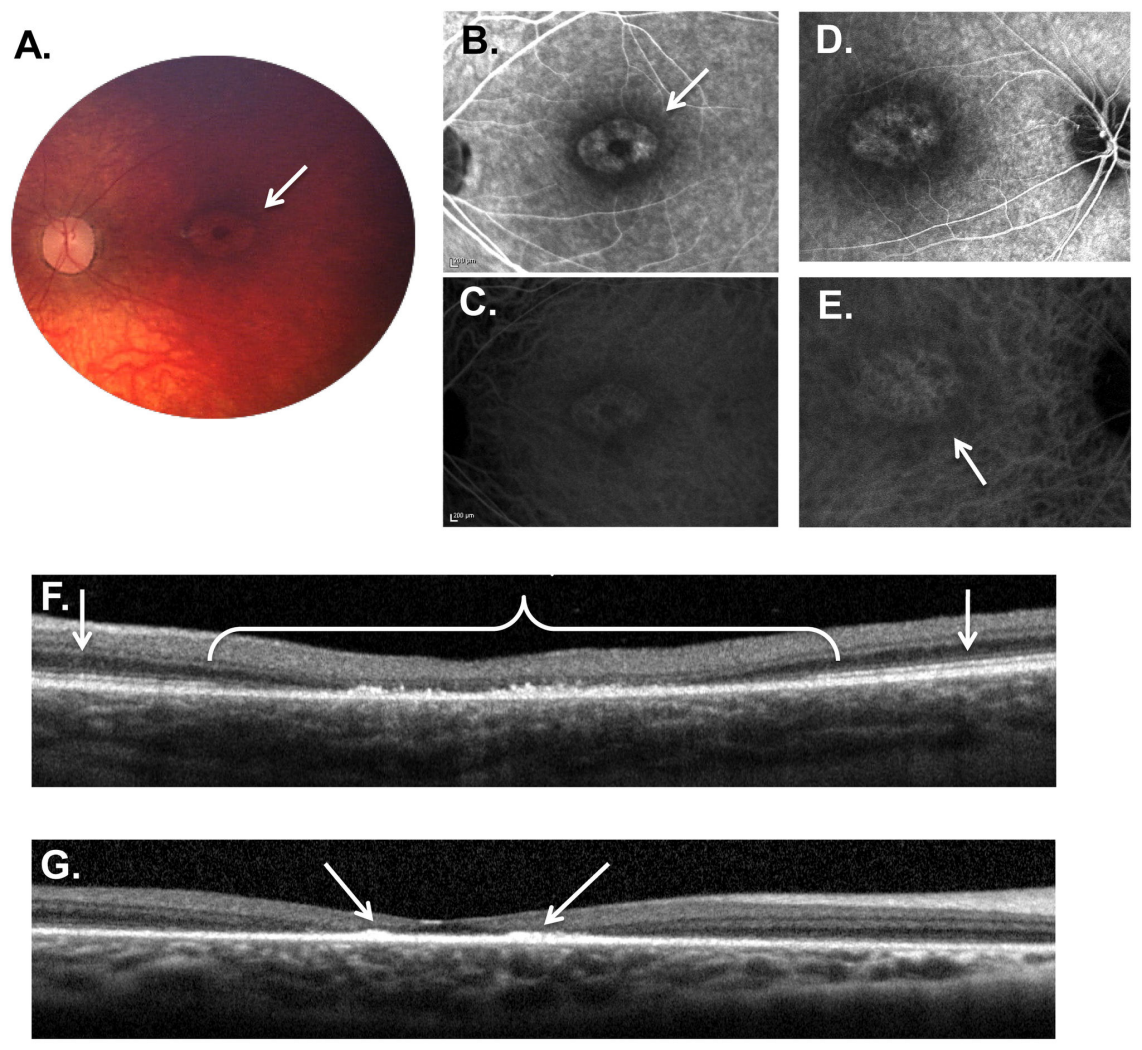
Weill Cornell LINCL Ophthalmic Severity Score 4. **A**. Dilated fundus photograph of patient 9 shows a “bull’s eye” maculopathy with concentric rings of pigmentary changes emanating from the fovea (arrow). Some optic nerve pallor with attenuation of retinal vessels is also noted. **B**. Late phase FA of the right eye of patient 9 showing the “bull’s eye” maculopathy. **C**. Late phase FA of the left eye of patient 8 demonstrates a similar pattern within the macula. **D**. Late phase ICGA of the right eye of patient 9 correlates with the FA. **E**. Late phase ICGA of the left eye of patient 8. **F**. SD-OCT of patient 8 demonstrates considerable outer retinal abnormalities (*), including outer retinal atrophy and buildup of hyper-reflective material at the level of the retinal pigment epithelium. This outer retinal disruption typically extends less than 2 disc diameters (*), with normal appearing retinal architecture beyond the central fovea (arrows). **G**. Interestingly, the hyper-reflective material in some patients (including patient 16 shown here), appears to preferentially accumulate in the immediate parafoveal region (arrows). The late-phase FA (**B** and **D**) and ICGA (**C** and **E**) images show the hyper- and hypo-fluorescence areas (arrows) corresponding to the pigmentary changes on the color photograph (A) and the outer retinal abnormalities noted on the SD-OCT (**F** and **G**). FA – fluorescein angiogram, ICGA - indocyanine green angiogram, SD-OCT – spectral domain optical coherence tomography.

**Figure 5:**
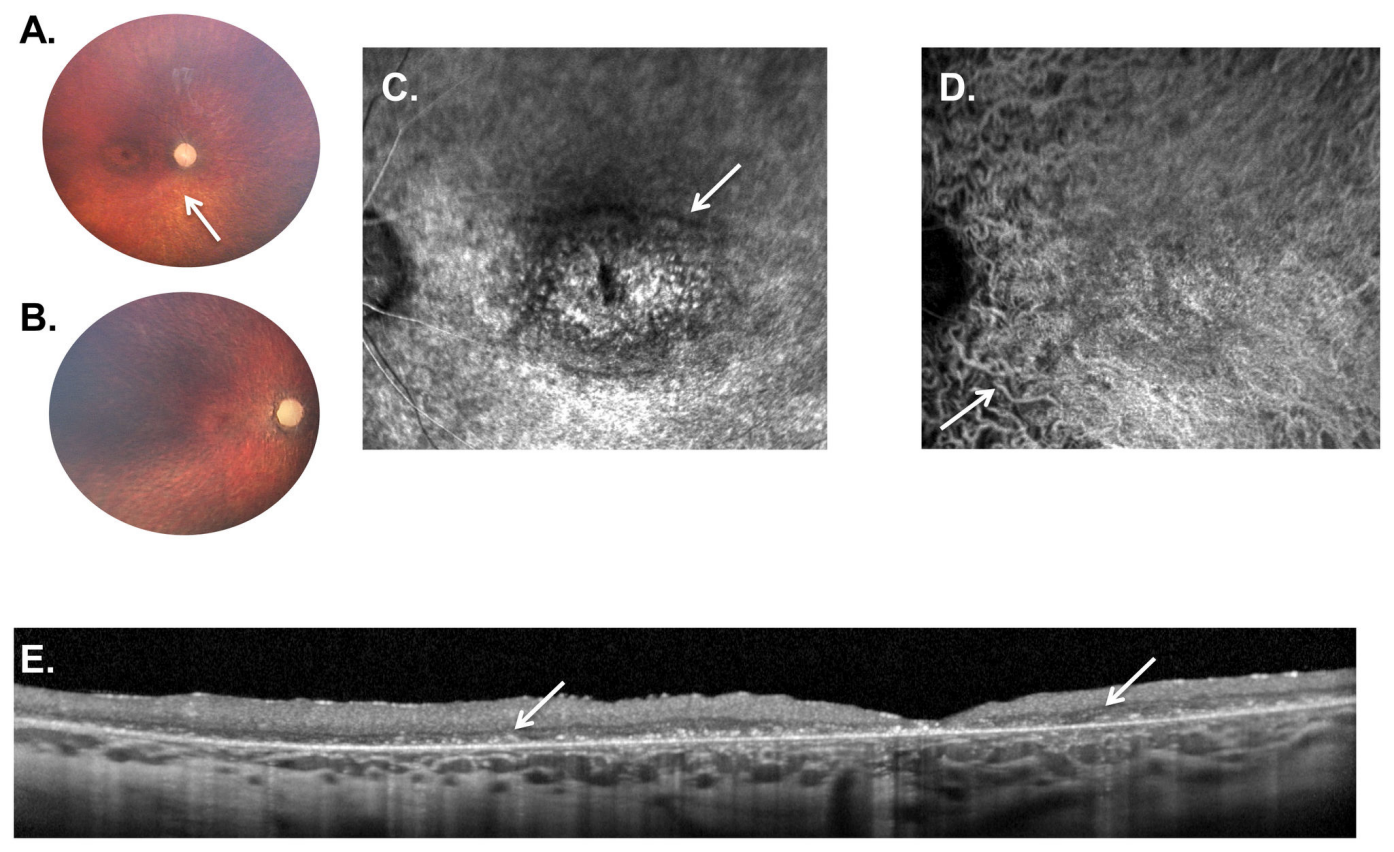
Weill Cornell LINCL Ophthalmic Severity Score 5. **A**. Dilated fundus photograph of patient 4 shows a “bull’s eye” maculopathy with concentric rings of pigmentary changes emanating from the fovea. The optic nerve is completely pale with severe attenuation of retinal vessels (arrow). **B**. In patient 19, with most severe neurological score in this series, the retina is completely atrophied with a pale optic nerve and barely recognizable retinal vessels. A “bull’s eye” maculopathy is not evident on the color photography in this severely affected patient. **C**. Late-phase FA and D. ICGA show a large “bull’s eye” maculopathy with concentric rings of hypo- and hyperfluorescence (arrow). Due to the retinal atrophy, the choroidal vessels are prominent throughout the ICGA (**D**, arrow). **E**. SD-OCT of patient 19 demonstrates considerable outer retinal atrophy with buildup of hyper-reflective material at the level of the retinal pigment epithelium. This outer retinal disruption extends throughout the entire fundus without normal appearing retinal architecture anywhere in the fovea. FA – fluorescein angiogram, ICGA - indocyanine green angiogram, SD-OCT – spectral domain optical coherence tomography.

There were no anterior segment abnormalities noted in any of the 50 eyes examined. All 50 eyes had white and quiet conjunctiva, a clear cornea, deep and quiet anterior chamber, flat iris, and clear lens. The vitreous cavity in all eyes appeared normal with an optically clear vitreous. Posterior segment findings ranged from a completely unremarkable dilated exam, OCT, FA, and ICGA to a severe, widespread retinal atrophy with complete absence of the outer retinal structures and the retinal pigment epithelium (RPE, Figures 1-5). The retinal degeneration was first noted in the macula, specifically in the parafoveal region, with subtle outer retinal and RPE abnormalities (Figure 2). These abnormalities then progressed from subtle pigmentary changes in the fovea (Figure 3) to a fulminant bull’s eye maculopathy, with a more widespread involvement of the outer retinal structures in the macula (Figure 4). In our series of LINCL patients, there was 100% correlation between the ophthalmic score in one eye and the ophthalmic score in the contralateral eye. The Supporting Information (Figure S1) shows representative images obtained on all 25 patients.

### LINCL Ophthalmic Severity Scale

Using the findings from ocular exam and ancillary testing, the Weill Cornell LINCL Ophthalmic Severity Scale was established (Table 2, Figures 1-5). A LINCL ophthalmic score of 1 represents a normal fundus exam (Table 2, Figure 1), while a score of 5 denotes nearly complete retinal atrophy in both the posterior pole and the peripheral retina (Table 2, Figure 5).

**Table 2:** Weill Cornell Late Infantile Neuronal Ceroid Lipofuscinosis (LINCL) Ophthalmic Severity Scale.

**Ophthalmic severity score**	**Color/fundus**	**Optical coherence tomography (OCT)**	**Fluorescein and indocyanine green angiogram and (FA/ICGA)**
1	*Optic Nerve* - normal	Normal retinal layers	Normal FA/ICGA without leakage or staining
	*Vasculature* - normal		
	*Macula* - normal		
	*Periphery* - normal		
2	*Optic Nerve* - normal	Normal retinal layers immediately adjacent at fovea; immediately outside fovea, outer retinal/photoreceptor atrophy	Subtle hyperfluorescence at fovea surrounded by ring of hypofluorescence extending less than 0.5 disc diameters from fovea
	*Vasculature* - normal		
	*Macula* - subtle pigmentary changes in parafoveal region extending less than 0.5 disc diameter		
	*Periphery* - normal		
3	*Optic Nerve* - normal to minimal pallor	Outer retinal atrophy/photoreceptor loss extending to 1.0 disc diameter from fovea; retinal layers appear normal outside fovea	Hypofluorescence in fovea surrounded by a ring of hyperfluorescence extending less than 1.0 disc diameters
	*Vasculature* - normal to minimal attenuation		
	*Macula* - small bull's eye pigmentary changes extending less than 1.0 disc diameter from fovea		
	*Periphery* - normal		
4	*Optic Nerve - m*ild to moderate pallor	Early buildup of outer retinal hyper-reflective material in macula; more extensive outer retinal abnormalities extending 1.0 to 2.0 disc diameters from fovea; beyond 2.0 disc diameters, outer retina appears normal	Bull's eye window defect (hyper- and hypofluorescence), extending between 1 to 2 disc diameters
	*Vasculature -* mild to moderate attenuation		
	*Macula* - bull's eye pigmentary changes extending more than 1.0 disc diameter from fovea		
	*Periphery -* pigmentary changes may be present		
5	*Optic Nerve* - severely pallor	Severe retinal thinning with generalized loss of photoreceptors and outer retina, involving the entire macula; extensive clumps of outer retinal hyper-reflective material; thickness map shows central thinning with paracentral thickening, with outer ring of further thinning (with bull's eye appearance); outer retinal changes also found outside the macula	Extensive (greater than 2 disc diameters) 360 degree bull's eye hyper- and hypofluorescence with late staining of outer retinal material noted on OCT; Speckled hypo- and hyperfluorescence outside of fovea
	*Vasculature* - severely attenuated		
	*Macula* - bull's eye maculopathy extending greater than 2 disc diameters		
	*Periphery* - retinal atrophy and pigmentary changes		

^1^ Patients underwent a comprehensive ophthalmic examination under anesthesia, including evaluation of the anterior segment, lens, and a dilated fundus exam. Color fundus photography, spectral domain optic coherence tomography, and fluorescein and indocyanine green angiography. Ophthalmic findings were grouped according to the severity of findings, with a score of 1 being the least severe and the score of 5, most severe. None of the patients exhibited any conjunctival, scleral, corneal, anterior chamber, iris or lenticular abnormalities.

#### Severity Score 1

A LINCL ophthalmic severity score of 1 showed a normal ophthalmic exam with no evidence of retinal degeneration either on exam or ancillary testing (Table 2, Figure 1). The fundus photograph, OCT, FA and ICGA all appeared within normal limits. In our cohort of LINCL patients, 8 of 25 patients (32.0%) had an ophthalmic severity score of 1 (Table 1). The average age of patients with severity score of 1 was 47.0 ± 11.7 months. This group of patients represented the largest proportion of LINCL eyes examined.

#### Severity Score 2

The fovea began to manifest subtle pigmentary changes in eyes with a LINCL severity score of 2 (Table 2, Figure 2). These outer retinal changes were noted in the parafoveal region and extended less than 0.5 disc diameters away from the fovea. The optic nerve, retinal vessels and the retinal periphery were all within normal limits. In our cohort, 6 of 25 patients (24.0%) had an ophthalmic severity score of 2 (Table 1). The average age of patients with severity score of 3 was 58.6 ± 5.8 months. Along with the ophthalmic score of 4, eyes with an ophthalmic score of 2 represented the second largest proportion of eyes examined, the score of 1 being the largest.

#### Severity Score 3

Eyes with a LINCL ophthalmic severity score of 3 showed mild to moderate pallor of the optic nerve, began to have retinal vascular attenuation, and exhibited a bull’s eye maculopathy that extended greater than 0.5 disc diameters, but less than 1 disc diameter away from the fovea (Table 2, Figure 3). OCT images showed outer retinal and photoreceptor loss corresponding to the pigmentary changes noted on the color fundus images. This abnormal outer retinal architecture was more extensive than that noted in eyes with an ophthalmic score of 2. FA and ICGA in these eyes demonstrated an area of hypofluorescence in fovea surrounded by a ring of hyperfluorescence that extended less than 1.0 disc diameter from the fovea. These angiogram abnormalities corresponded to the retinal irregularities noted on the OCT and color images. Outside this area of the bull’s eye maculopathy, the retina appeared normal, with normal outer retinal architecture on OCT. The periphery was also unaffected in eyes with a severity score of 3. Two of 25 patients (8.0%) had an ophthalmic severity score of 3 (Table 1), representing the smallest proportion of LINCL eyes examined. The average age of patients with severity score of 3 was 69.0 ± 14.1 months.

#### Severity Score 4

Eyes with a LINCL ophthalmic severity score of 4 revealed a more extensive retinal degeneration, although areas of normal retina, even in the posterior pole, still remained (Table 2, Figure 4). Findings on dilated fundus exam included mild to moderate pallor of the optic nerve, mild to moderate attenuation of the retinal vessels and pigmentary changes in a bull’s eye pattern that extended more than 1.0 disc diameter from fovea. Unlike the scores of lesser severity, these pigmentary changes were also present in the periphery of eyes with a severity score of 4. FA and ICGA in these patients showed a window defect and blocking, with areas of hyper- and hypofluorescence, extending between 1 to 2 disc diameters from the fovea in a bull’s eye pattern. Spectral domain OCT through the macula demonstrated an early buildup of outer retinal hyper-reflective material along with photoreceptor, RPE and outer retinal abnormalities that extended 1.0 to 2.0 disc diameters from fovea; beyond the approximately 2.0 disc diameters, the outer retinal architecture appeared normal. In our cohort of LINCL patients, 6 of 25 patients (24.0%) had an ophthalmic severity score of 4 (Table 1). The average age of patients with severity score of 4 was 61.2 ± 4.7 months. Along with an ophthalmic score of 2, eyes with an ophthalmic score of 4 represented the second largest proportion of eyes examined.

#### Severity Score 5

The most severe and widespread retinal abnormalities were noted in patients with a LINCL ophthalmic severity score of 5 (Table 2, Figure 5). Patients with a severity score of 5 had pale optic nerves with severely attenuated retinal vessels. A bull’s eye maculopathy extended greater than 2 disc diameters from the fovea. Pigmentary changes were noted throughout the posterior pole as well as in the peripheral retina. Spectral domain OCT demonstrated severe retinal thinning, generalized loss of photoreceptors and outer retina, and extensive clumps of outer retinal hyper-reflective material, along with RPE atrophy. These outer retinal changes were also evident in OCT images performed outside the macula. Findings on FA and ICGA paralleled those noted on fundus photography and OCT, with severely attenuated vessels and areas of speckled hypo- and hyperfluorescence noted throughout the fundus (in a bull’s eye pattern in the macula). In our cohort of LINCL patients, 3 of 25 patients (12.0%) had an ophthalmic severity score of 5 (Table 1). The average age of patients with severity score of 5 was 81.0 ± 17.1 months. These patients made up the second smallest proportion of LINCL eyes in the cohort.

### Correlation of the Ophthalmologic Score with Other Parameters

**Figure 6:**
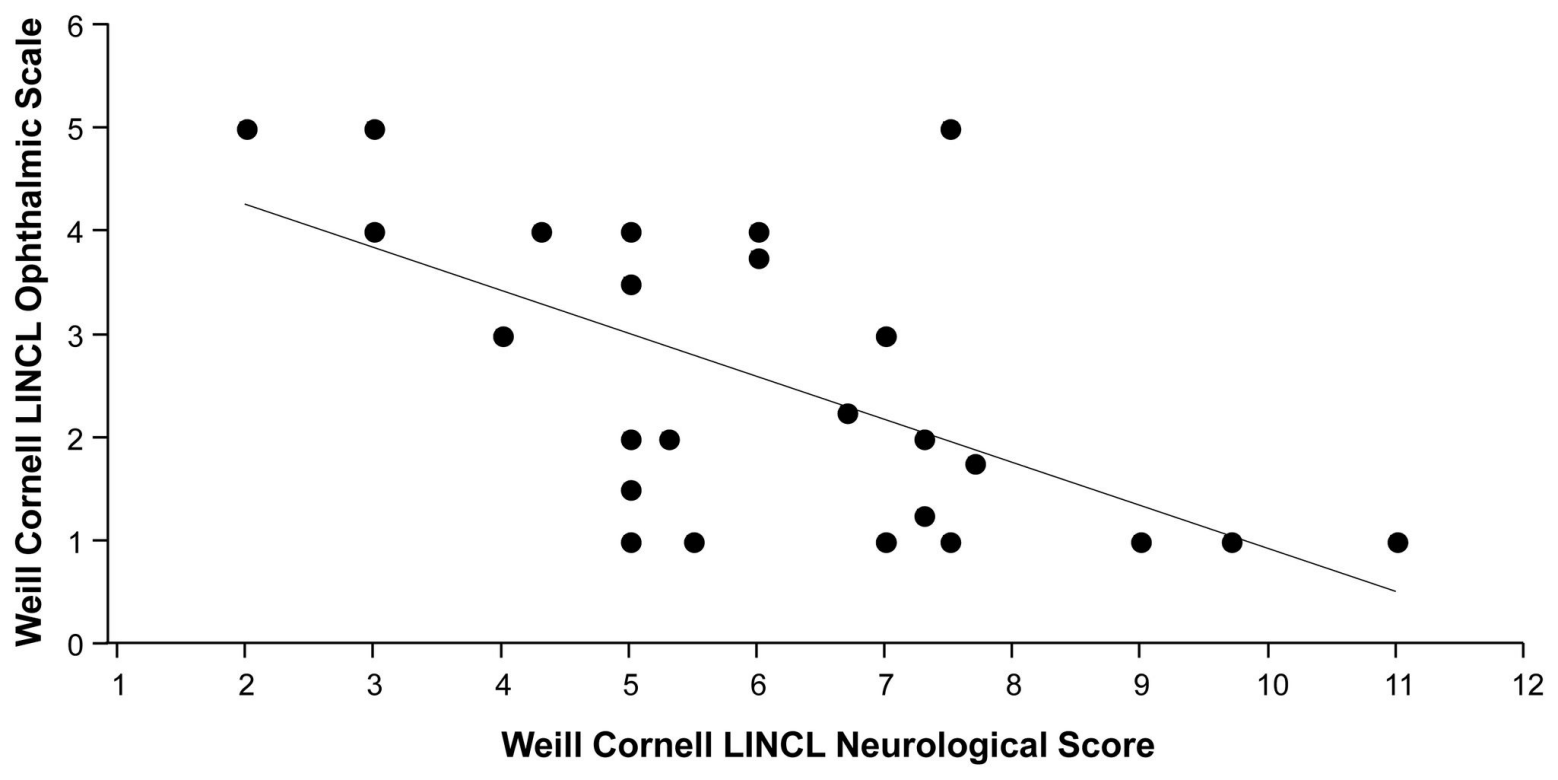
Association of Weill Cornell LINCL Ophthalmic Severity Scale and Weill Cornell LINCL Neurological Score. A direct correlation was found between this new Weill Cornell LINCL Ophthalmic Severity Scale and the previously validated neurological scale (Kendall’s tau = -0.476, p<0.002). More severe ophthalmic manifestations were observed in patients with more severe neurological findings (Kruskal-Wallis Test: X^2^ = 4.8, p<0.03).

**Figure 7:**
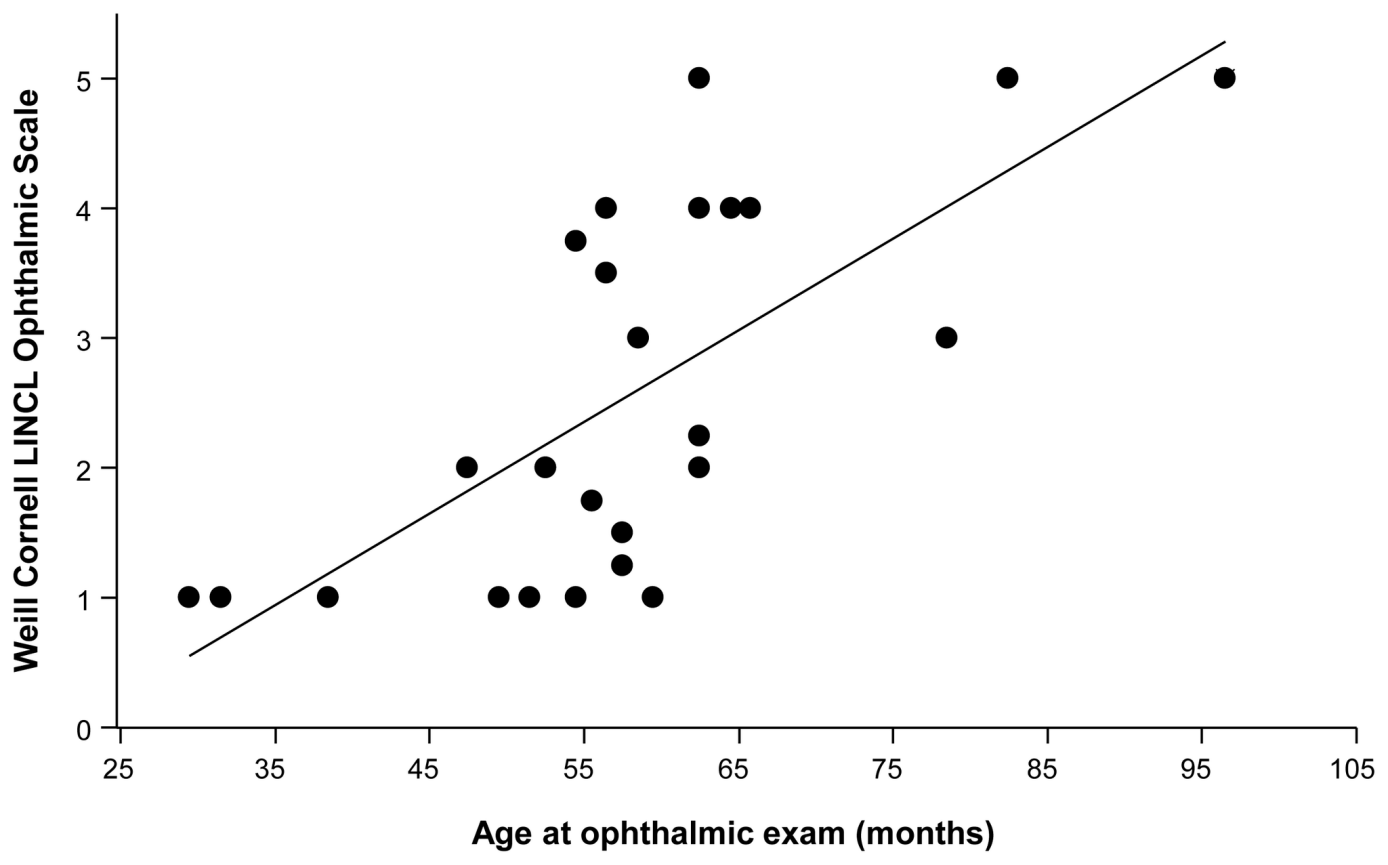
Correlation of Weill Cornell LINCL Ophthalmic Severity Scale and Age. A direct association was found between age and the Weill Cornell LINCL Ophthalmic Severity Scale (Kendall’s tau =3.79, p<0.0002), with older patients having more severe ophthalmic manifestations (Kruskal-Wallis Test: X^2^ = 8.2, p<0.005).

The mean LINCL ophthalmic severity score was 2.6 (median -2.0, mode -1.0, range 1 to 5, Table 1). An excellent correlation was found among the four retina specialists for determination of each patient’s LINCL ophthalmic severity score, where all correlations were highly significant (all Kendall’s tau > 0.87, p<0.0001). More severe ophthalmic manifestations were observed among patients with worse neurological findings (Kruskal-Wallis Test: X^2^ = 4.8, p<0.03, Figure 6) and among older patients (Kruskal-Wallis Test: X^2^ = 8.2, p<0.005, Figure 7). A direct correlation was found between this new Ophthalmic Severity Scale and the previously validated neurological scale (Kendall’s tau = -0.476, p<0.002, Figure 6). A direct association was also found between age and the Ophthalmic Severity Scale (Kendall’s tau =3.79, p<0.0002, Figure 7), with older patients having more severe ophthalmic manifestations.

## Discussion

Neuronal ceroid lipofuscinoses are a heterogeneous group of lysosomal storage diseases characterized by a progressive neurodegenerative process resulting in loss of vision, seizures, movement disorders, mental regression, behavioral abnormalities, and a shortened life expectancy [1–4]. In the present study, we have systematically categorized the ophthalmic findings of LINCL and correlated the ocular abnormalities with clinical parameters.

We describe and classify the ophthalmic manifestations of LINCL in 50 eyes of 25 patients with genetically confirmed *CLN2* mutations. The ocular findings are typified by a gradually progressive retinal degeneration, commencing at the level of the outer retina (specifically in the RPE and the photoreceptors, in a bull’s eye pattern) and progressing from the central macula to the peripheral retina. In the most severe cases, this retinal degeneration ultimately results in widespread retinal atrophy encompassing the entire fundus. Interestingly, none of the eyes in our cohort exhibited any anterior segment abnormalities, regardless of the severity of the retinal degeneration, the advancing age of the patient or the extent of neurological deterioration, which is in contrast to other lysosomal storage diseases that can manifest with anterior segment findings such as corneal opacities [26,27].

Based on the findings of the ophthalmic exam and the ancillary ophthalmic testing (including FA, ICGA, and OCT), we developed a five step Weill Cornell LINCL Ophthalmic Severity Scale (score of 1 to 5, from least to most severe retinal findings). Eyes with a LINCL score of 1 show no apparent ophthalmic abnormalities. This is in direct contrast to the eyes with a severity score of 5 that have extensive retinal degeneration and widespread retinal atrophy. The Weill Cornell LINCL Ophthalmic Severity Scale was easily comprehended and learned by our ophthalmology colleagues as evidenced by the high degree of correlation among the 4 independent masked graders.

The ophthalmic findings noted in our cohort of patients are consistent with the few previous reports of ocular manifestations of LINCL [17–25]. These descriptions of LINCL patients are typically part of larger NCL cohorts, and usually include the fundus examination with limited and variable ancillary testing. To the best of our knowledge, this current cohort is the largest series of LINCL patients who have had prospectively performed, standardized, comprehensive ophthalmic evaluations and neurological assessments, and the only cohort of NCL patients to have had ICGA and spectral domain OCT evaluations. Although the ICGA may add little in terms of diagnosis or classification of severity beyond what is noted on the FA, the progression of outer retinal atrophy and pigmentary changes is easily recognizable on the high-resolution spectral-domain OCT images. Even very early subtle changes can be demonstrated on the OCT, most impressively in the eyes with a LINCL ophthalmic score of 2 that show subtle parafoveal outer retinal changes. A dilated fundus exam and a macular high-resolution OCT may, in the end, be adequate to establish the extent and to follow the progression of retinal abnormalities in LINCL patients.

A previously published report on the electron microscopic findings in a patient with LINCL confirms our clinical findings that the retinal destruction in this disease starts at the photoreceptor and outer retinal levels and progresses from the macular area to the periphery [24]. The microscopic findings noted in this report are comparable to that seen on the high-resolution spectral-domain OCT images in our patients. The outer retinal (photoreceptor and RPE) atrophy is also similar to that noted in two reports of JNCL eyes evaluated at autopsy [17,19].

Our findings are also consistent with a prior description by Hainsworth and colleagues [25] of the funduscopic and angiographic appearance of NCL patients. In their entire cohort of NCL patients, Hainsworth noted optic nerve pallor, vascular attenuation, RPE atrophy, orange pigment clumping, and epiretinal membrane in the macula, along with bone spicules, RPE atrophy and course pigment stippling in the periphery. Of the 29 patients in the report, 24 were diagnosed with JNCL and 2 with LINCL [25]. These 2 LINCL patients were evaluated with color fundus photography alone. None of the 29 patients in this previous report had a systematic neurological assessment. As such, correlation of the extent of ophthalmic findings with NCL disease progression was not possible.

The optic nerve pallor, vascular attenuation, macular atrophy and pigmentary changes reported by Kelly and colleagues [22] in their cohort of 27 NCL patients are also consistent with our description of ocular findings in LINCL. In addition to color fundus photography, in this cohort, 9 NCL patients underwent standardized fundus autofluorescence (FAF) imaging. Kelly and colleagues [22] report an increased FAF early in the disease course and decreased FAF with more extensive retinal atrophy. The exact subset of LINCL patients who were imaged is not specified in the report, nor is the extent of disease progression noted in any of the NCL patients examined. The 25 patients in our current cohort did not undergo standardized FAF imaging, and as such, any possible FAF abnormalities were not incorporated into the Ophthalmic Severity Scale.

A distinctive aspect of our report is the integration of the extent of neurological worsening with the findings noted on ophthalmic evaluation. Not surprisingly, in the 25 patients evaluated in this study, there was a high degree of correlation between the Weill Cornell LINCL Ophthalmic Scale and the Weill Cornell LINCL neurological scale, with more progressive retinal degeneration noted in patients with worse neurological deterioration. All patients exhibited at least some extent of neurological dysfunction as determined by the Weill Cornell LINCL neurological scale. The youngest patient was the most neurologically intact, with the best neurological score and the best ophthalmic score. The oldest patient had the most progressive neurological deterioration in the entire cohort, and exhibited widespread retinal degeneration. Interestingly, Kendall’s tau for correlation between ophthalmic and neurological scores, while significant, was not exceedingly high, suggesting that the two scores may describe similar but not identical parameters. This may be due, in part, to the fact that the neurological score represents an aggregate of feeding, gait, motor and language abilities and thus defines a more global neurological deterioration noted in LINCL patients. The ophthalmic score, on the other hand, is specific to the progressive degeneration in a particular part of the CNS, that of the eye. Moreover, the ophthalmic testing (in particular the OCT, FA and ICG) may be more sensitive than gross motor and cognitive skills to subtle changes associated with the progressive degeneration in LINCL. It is also possible that the motor and language skills reflected in the neurological score have a slightly differing degenerative course compared with that of the ophthalmic findings reported here.

In a prior study, Steinfeld et al [14] developed a neurological rating scale to assess LINCL disease progression (e.g., neurological score). This scale had a visual ability, where a patient’s visual function (ranging from recognition and grabbing of an object to no light perception) was incorporated into the overall LINCL scoring system. Although this was a useful initial step, neither an ophthalmic examination nor any ancillary ophthalmic testing was a part of this study. To the best of our knowledge, there has been no previous attempt to correlate the progression of LINCL with that of the ocular findings that include a full ophthalmic examination and a battery of ophthalmic tests.

One limitation of our study is the lack of ophthalmic evaluations in awake LINCL patients. The already apparent mental regression and gross motor disturbances evident even in the least severely affected LINCL patients in our cohort precluded any accurate evaluation of visual function (e.g. visual acuity, visual field) or ophthalmic examination in an outpatient unsedated setting. We attempted to examine the first 4 LINCL patients in our outpatient ophthalmology clinic. However, we could not reliably perform visual acuity assessment or even a complete dilated fundus exam. Ancillary testing (OCT, FA, ICGA) in this setting was impossible in any of these 4 LINCL patients. Therefore, all 25 patients were examined and ancillary testing was performed under anesthesia. The visual acuity assessment in the Hamburg LINCL scale mirrors our experience with the difficultly in accurately determining visual function in these patients (the visual score in this scale ranges from recognition and grabbing at an object, uncoordinated grabbing, reaction to light, and finally no reaction to visual stimuli) [14].

Given the context of a gene therapy clinical trial from which all the patients were recruited, our cohort is biased toward less severely affected LINCL patients. As such, a majority of the eyes (28 of 50 eyes, 56%) in the current study demonstrated either no identifiable ophthalmic manifestations (Severity Score of 1) or very subtle changes that were noted primarily on ancillary ophthalmic testing (Severity Score of 2). These patients may be the ones who might ultimately gain the most clinical benefit from possible LINCL treatments for its ophthalmic manifestations, especially prior to the onset of severe widespread retinal degeneration. Moreover, response to any therapy for LINCL can easily be monitored in a relatively non-invasive fashion with serial ophthalmic evaluations.

As the patients who were included in this current manuscript were undergoing ophthalmic evaluations as part of a larger gene therapy trial, the availability of specific ancillary testing devices varied slightly between patients. The primary purpose of the ocular examinations was to screen for inclusion in the CNS gene therapy study rather than for the development of an ophthalmic scale. All patients were evaluated with a comprehensive dilated fundus exam, dilated fundus photography and FA. With the availability of specific equipment in the operating room, 68% of patients also had an OCT and 60% an ICGA. FA, ICGA and OCT each provide slightly different but complementary information about the retina. As typical for other ophthalmic disorders, abnormalities on OCT can also be observed on FA. The advantage of an OCT is that it is non-invasive, with no requirement for injection of a fluorescein dye to complete the testing. As LINCL changes appear to be predominantly retinal in nature, ICGA, typically utilized for detecting changes in the choroid, may not be important for the ophthalmic severity determination. Therefore, based on our experience, the most important ophthalmic examinations for evaluating the extent of disease severity included a dilated fundus exam and an OCT. FA and ICGA may not be necessary in all cases. There is, however, a possibility that the availability of OCT and ICGA for all the patients may have influenced the ophthalmic severity score reported in this current study.

In the current study, we describe the ophthalmic manifestations of LINCL, develop an objective and easily comprehensible ophthalmic LINCL severity scale, and correlate the ophthalmic manifestations with the extent of neurological decline and advancing age of patients with *CLN2* mutations. This newly established Ophthalmic Scale may serve as an independent clinical indicator of LINCL severity and disease progression. Our multimodality approach of using a dilated exam, combined with FA, ICGA, and OCT and correlating these with the systemic manifestations may also be applied more broadly to characterize the ocular manifestations of other inherited lysosomal storage disorders with known ophthalmic findings (e.g., Niemann–Pick disease, Tay–Sachs disease, Sandhoff disease, and others). These ophthalmic manifestations may ultimately be beneficial in the assessment of novel therapeutic approaches for the treatment lysosomal storage disorders, such as stem cell and gene therapy, directed either systemically or locally at the central nervous system or the eye.

## Supporting Information

Figure S1 **Representative images from the 50 eyes of the 25 patients with LINCL.**
Patients 1, 2, 5 and 21-25 underwent dilated fundus photography and FA; patients 3 and 6 had dilated fundus photography, FA and SD-OCT; patients 4, and 7-20 underwent the most comprehensive evaluation with dilated color photography, FA, ICGA and SD-OCT. FA – fluorescein angiogram, ICGA – indocyanine green angiogram, SD-OCT – spectral domain optical coherence tomography.
(PDF)

## References

[1] Boustany RM (1996) Batten disease or neuronal ceroid lipofuscinosis. In: Moser HW Handbook of Clinical Neurology. Elsevier Science pp. 671-700.

[2] Haltia M (2006) The neuronal ceroid-lipofuscinoses: from past to present. Biochim Biophys Acta 1762: 850-856. doi:10.1016/j.bbadis.2006.06.010. PubMed: 16908122. 16908122 10.1016/j.bbadis.2006.06.010

[3] Kohlschütter A, Schulz A (2009) Towards understanding the neuronal ceroid lipofuscinoses. Brain Dev 31: 499-502. doi:10.1016/j.braindev.2008.12.008. PubMed: 19195801. 19195801 10.1016/j.braindev.2008.12.008

[4] Jalanko A, Braulke T (2009) Neuronal ceroid lipofuscinoses. Biochim Biophys Acta 1793: 697-709. doi:10.1016/j.bbamcr.2008.11.004. PubMed: 19084560. 19084560 10.1016/j.bbamcr.2008.11.004

[5] Kousi M, Lehesjoki AE, Mole SE (2012) Update of the mutation spectrum and clinical correlations of over 360 mutations in eight genes that underlie the neuronal ceroid lipofuscinoses. Hum Mutat 33: 42-63. doi:10.1002/humu.21624. PubMed: 21990111. 21990111 10.1002/humu.21624

[6] Palmer DN, Fearnley IM, Walker JE, Hall NA, Lake BD et al. (1992) Mitochondrial ATP synthase subunit c storage in the ceroid-lipofuscinoses (Batten disease). Am J Med Genet 42: 561-567. doi:10.1002/ajmg.1320420428. PubMed: 1535179. 1535179 10.1002/ajmg.1320420428

[7] Palmer DN, Jolly RD, van Mil HC, Tyynelä J, Westlake VJ (1997) Different patterns of hydrophobic protein storage in different forms of neuronal ceroid lipofuscinosis (NCL, Batten disease). Neuropediatrics 28: 45-48. doi:10.1055/s-2007-973666. PubMed: 9151321. 9151321 10.1055/s-2007-973666

[8] Williams RE, Gottlob I, Lake BD, Goebel HH, Winchester BG et al. (1999) Classic late infantile NCL. In: Goebel HH The Neuronal Ceroid Lipofuscinosis (Batten Disease). IOS Press pp. 37-54.

[9] Sleat DE, Donnelly RJ, Lackland H, Liu CG, Sohar I et al. (1997) Association of mutations in a lysosomal protein with classical late-infantile neuronal ceroid lipofuscinosis. Science 277: 1802-1805. doi:10.1126/science.277.5333.1802. PubMed: 9295267. 9295267 10.1126/science.277.5333.1802

[10] Sleat DE, Gin RM, Sohar I, Wisniewski K, Sklower-Brooks S et al. (1999) Mutational analysis of the defective protease in classic late-infantile neuronal ceroid lipofuscinosis, a neurodegenerative lysosomal storage disorder. Am J Hum Genet 64: 1511-1523. doi:10.1086/302427. PubMed: 10330339. 10330339 10.1086/302427 PMC1377895

[11] Vines DJ, Warburton MJ (1999) Classical late infantile neuronal ceroid lipofuscinosis fibroblasts are deficient in lysosomal tripeptidyl peptidase I. FEBS Lett 443: 131-135. doi:10.1016/S0014-5793(98)01683-4. PubMed: 9989590. 9989590 10.1016/s0014-5793(98)01683-4

[12] Rawlings ND, Barrett AJ (1999) Tripeptidyl-peptidase I is apparently the CLN2 protein absent in classical late-infantile neuronal ceroid lipofuscinosis. Biochim Biophys Acta 1429: 496-500. doi:10.1016/S0167-4838(98)00238-6. PubMed: 9989235. 9989235 10.1016/s0167-4838(98)00238-6

[13] Seehafer SS, Pearce DA (2006) You say lipofuscin, we say ceroid: defining autofluorescent storage material. Neurobiol Aging 27: 576-588. doi:10.1016/j.neurobiolaging.2005.12.006. PubMed: 16455164. 16455164 10.1016/j.neurobiolaging.2005.12.006

[14] Steinfeld R, Heim P, von GH, Meyer K, Ullrich K et al. (2002) Late infantile neuronal ceroid lipofuscinosis: quantitative description of the clinical course in patients with CLN2 mutations. Am J Med Genet 112: 347-354. doi:10.1002/ajmg.10660. PubMed: 12376936. 12376936 10.1002/ajmg.10660

[15] Worgall S, Kekatpure MV, Heier L, Ballon D, Dyke JP et al. (2007) Neurological deterioration in late infantile neuronal ceroid lipofuscinosis. Neurology 69: 521-535. doi:10.1212/01.wnl.0000267885.47092.40. PubMed: 17679671. 17679671 10.1212/01.wnl.0000267885.47092.40

[16] Bozorg S, Ramirez-Montealegre D, Chung M, Pearce DA (2009) Juvenile neuronal ceroid lipofuscinosis (JNCL) and the eye. Surv Ophthalmol 54: 463-471. doi:10.1016/j.survophthal.2009.04.007. PubMed: 19539834. 19539834 10.1016/j.survophthal.2009.04.007 PMC4139962

[17] Goebel HH, Klein H, Santavuori P, Sainio K (1988) Ultrastructural studies of the retina in infantile neuronal ceroid-lipofuscinosis. Retina 8: 59-66. doi:10.1097/00006982-198808010-00011. PubMed: 3406546. 3406546 10.1097/00006982-198808010-00011

[18] Weleber RG, Gupta N, Trzupek KM, Wepner MS, Kurz DE et al. (2004) Electroretinographic and clinicopathologic correlations of retinal dysfunction in infantile neuronal ceroid lipofuscinosis (infantile Batten disease). Mol Genet Metab 83: 128-137. doi:10.1016/j.ymgme.2004.06.019. PubMed: 15464427. 15464427 10.1016/j.ymgme.2004.06.019

[19] Bensaoula T, Shibuya H, Katz ML, Smith JE, Johnson GS et al. (2000) Histopathologic and immunocytochemical analysis of the retina and ocular tissues in Batten disease. Ophthalmology 107: 1746-1753. doi:10.1016/S0161-6420(00)00264-5. PubMed: 10964839. 10964839 10.1016/s0161-6420(00)00264-5

[20] Puga AC, Jardim LB, Chimelli L, De Souza CF, Clivati M (2000) Neuronal ceroid lipofuscinoses: a clinical and morphological study of 17 patients from southern Brazil. Arq Neuro Psiquiatr 58: 597-606. doi:10.1590/S0004-282X2000000400001. PubMed: 10973097. 10.1590/s0004-282x2000000400001 10973097

[21] Collins J, Holder GE, Herbert H, Adams GG (2006) Batten disease: features to facilitate early diagnosis. Br J Ophthalmol 90: 1119-1124. doi:10.1136/bjo.2006.091637. PubMed: 16754648. 16754648 10.1136/bjo.2006.091637 PMC1857407

[22] Kelly JP, Weiss AH, Rowell G, Seigel GM (2009) Autofluorescence and infrared retinal imaging in patients and obligate carriers with neuronal ceroid lipofuscinosis. Ophthal Genet 30: 190-198. doi:10.3109/13816810903258829. PubMed: 19852577. 10.3109/13816810903258829 19852577

[23] Weleber RG (1998) The dystrophic retina in multisystem disorders: the electroretinogram in neuronal ceroid lipofuscinoses. Eye (Lond) 12 (Pt 3b): 580-590 PubMed: 9775220. 10.1038/eye.1998.148 9775220

[24] Traboulsi EI, Green WR, Luckenbach MW, de la Cruz ZC (1987) Neuronal ceroid lipofuscinosis. Ocular histopathologic and electron microscopic studies in the late infantile, juvenile, and adult forms. Graefes Arch Clin Exp Ophthalmol 225: 391-402. doi:10.1007/BF02334164. PubMed: 3678849. 3678849 10.1007/BF02334164

[25] Hainsworth DP, Liu GT, Hamm CW, Katz ML (2009) Funduscopic and angiographic appearance in the neuronal ceroid lipofuscinoses. Retina 29: 657-668. doi:10.1097/IAE.0b013e31819b0542. PubMed: 19289983. 19289983 10.1097/IAE.0b013e31819b0542

[26] Biswas J, Nandi K, Sridharan S, Ranjan P (2008) Ocular manifestation of storage diseases. Curr Opin Ophthalmol 19: 507-511. doi:10.1097/ICU.0b013e32831215c3. PubMed: 18854696. 18854696 10.1097/ICU.0b013e32831215c3

[27] Lanzl I, Leroy B (2008) Ocular features of treatable lysosomal storage disorders- Fabry Disease, mucopolysaccharidoses I, II and VI, and Gaucher Disease. U S Ophthalmic Rev: 44-51.

