# Supplementary material for: Spectrum of Ocular Manifestations in CLN2-Associated Batten (Jansky-Bielschowsky) Disease Correlate with Advancing Age and Deteriorating Neurological Function
Source: PLoS One. 2013 Aug 28;8(8):e73128. doi: 10.1371/journal.pone.0073128 (PMC3756041; doi:10.1371/journal.pone.0073128)

Supplemental Figure Legend. Representative images from the 50 eyes of the 25 patients with LINCL. Patients 1, 2, 5 and 21 – 25 underwent dilated fundus photography and FA; Patients 3 and 6 had dilated fundus photography, FA and SD-OCT; Patients 4, 7 – 20 underwent the most comprehensive evaluation with dilated color photography, FA, ICGA and SD-OCT. FA – fluorescein angiogram, ICGA - indocyanine green angiogram, SD-OCT – spectral domain optical coherence tomography.

Subject 1 – BDrh-01-IW – Color, FA

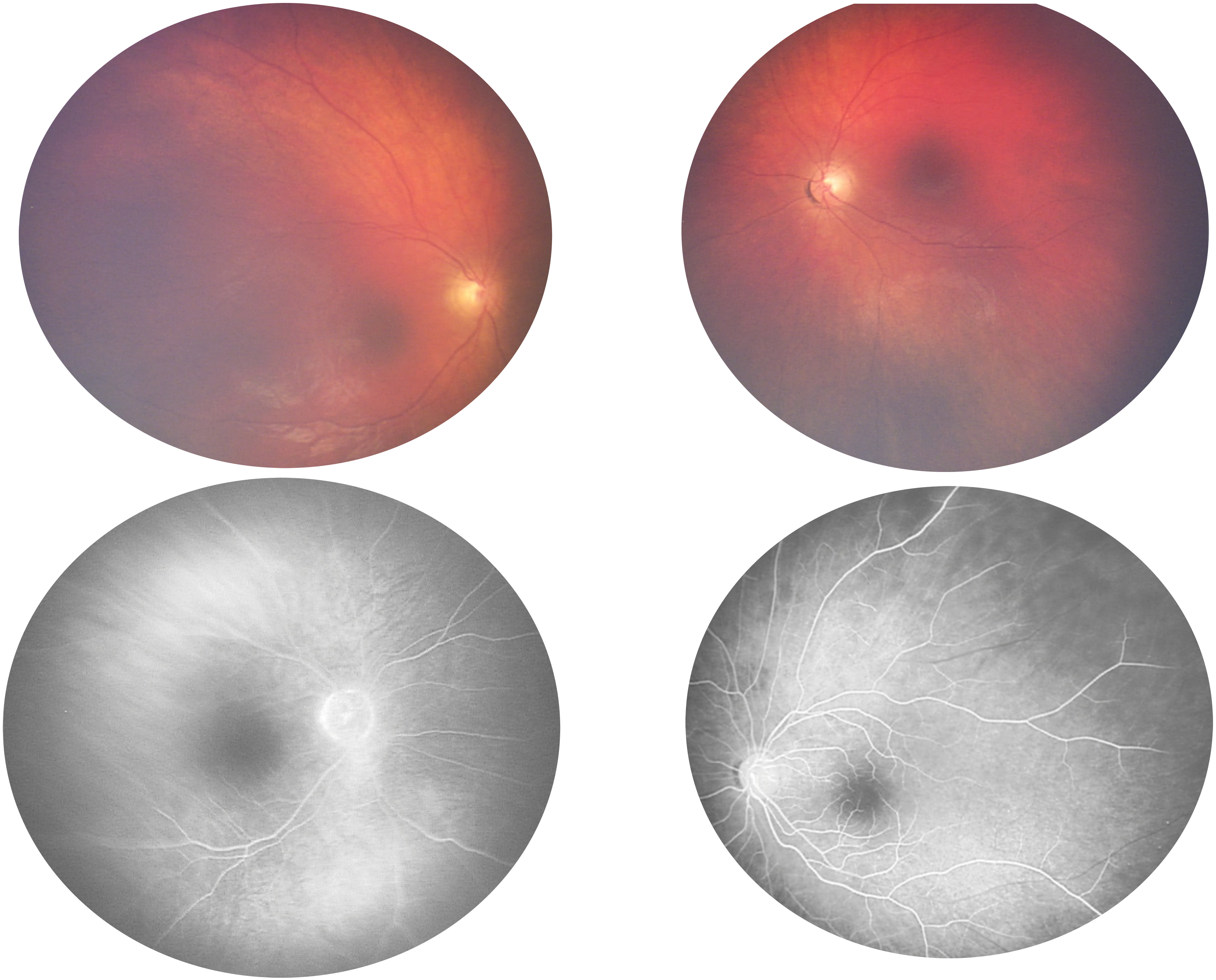

Subject 2 – BDrh-02-IN – Color, FA

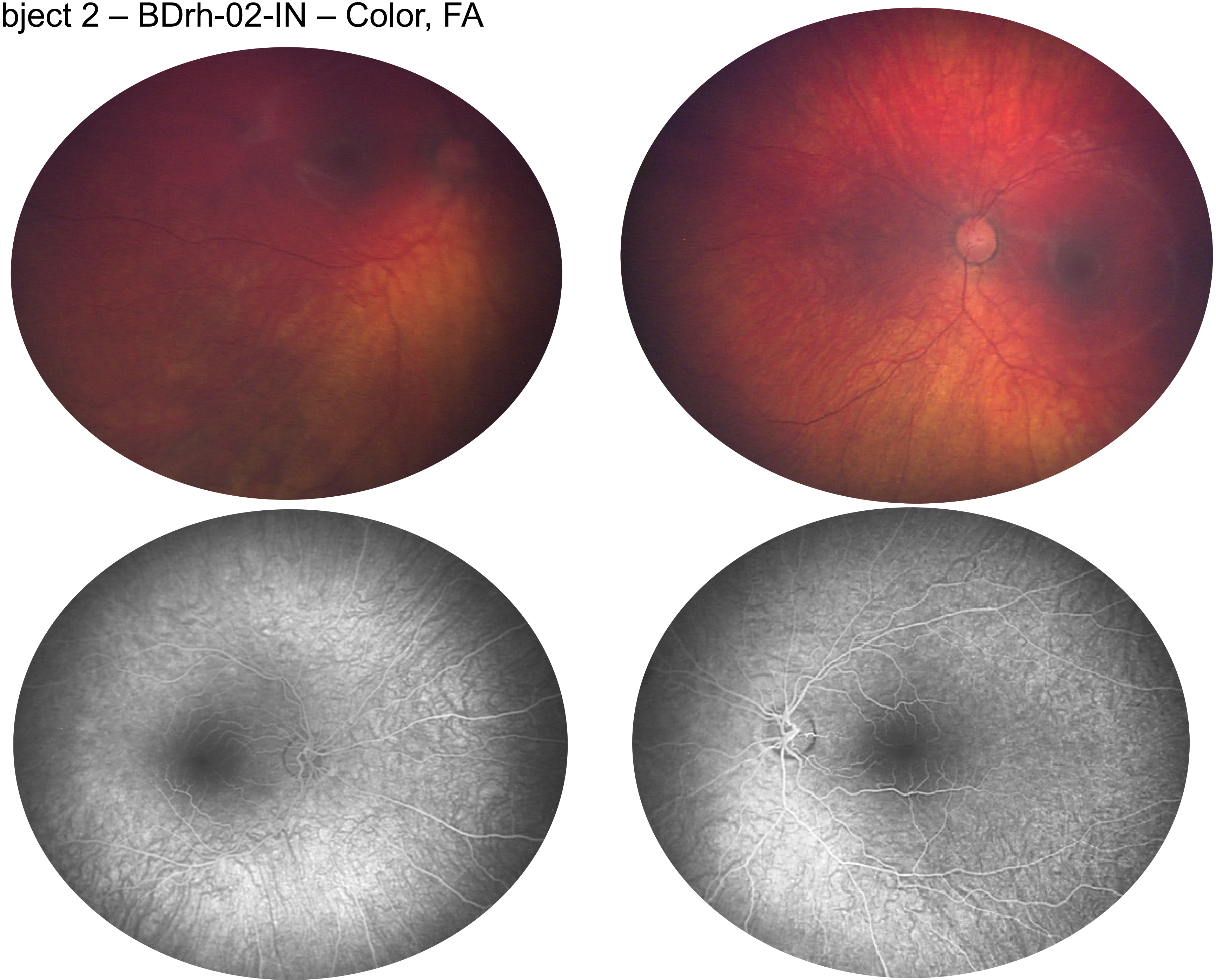

Subject 3 – BDrh-03-CI – Color, FA, OCT

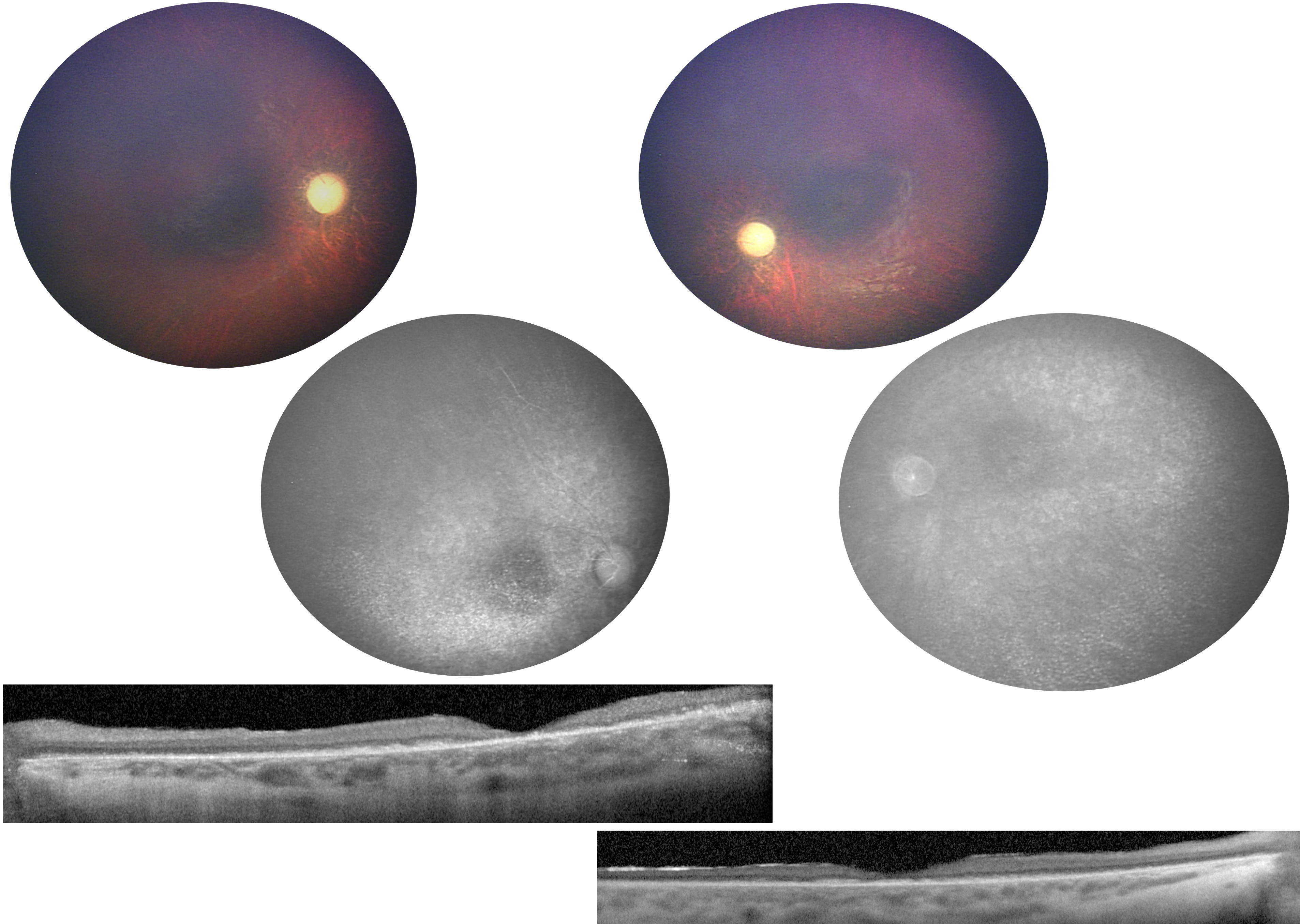

Subject 4 – BDrh-04-SI – Color, FA/ICGA, OCT

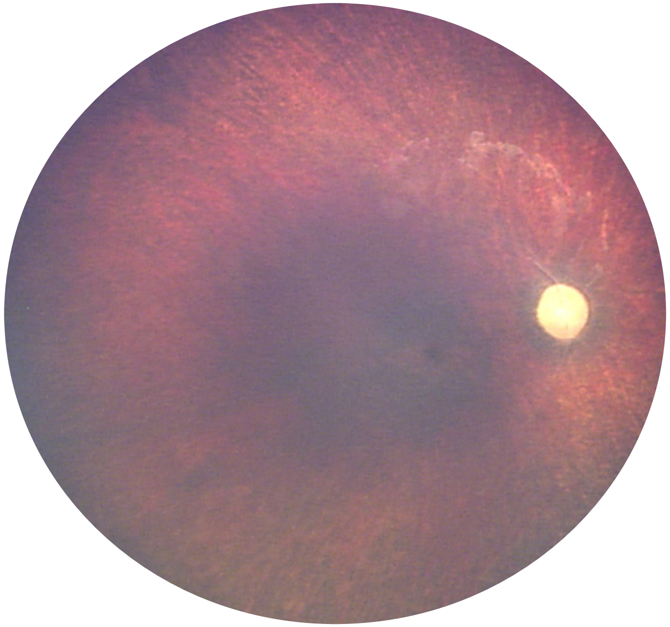

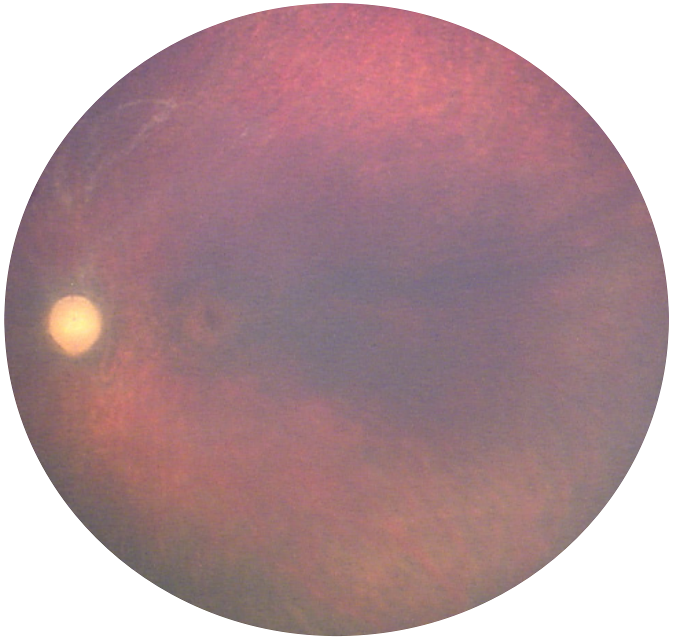

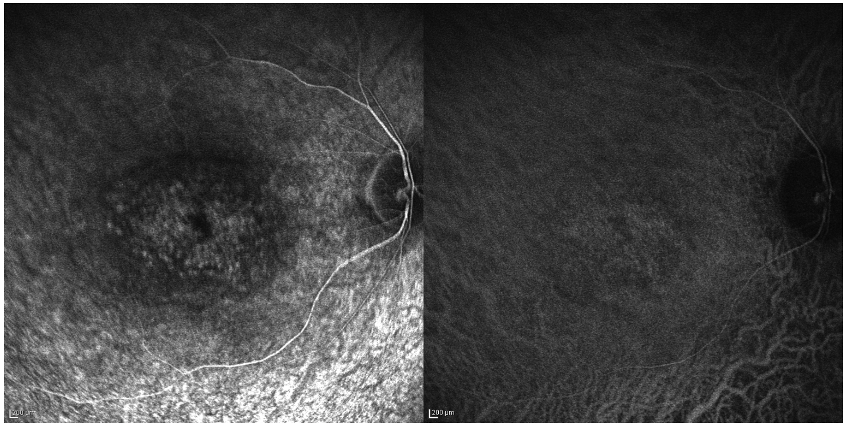

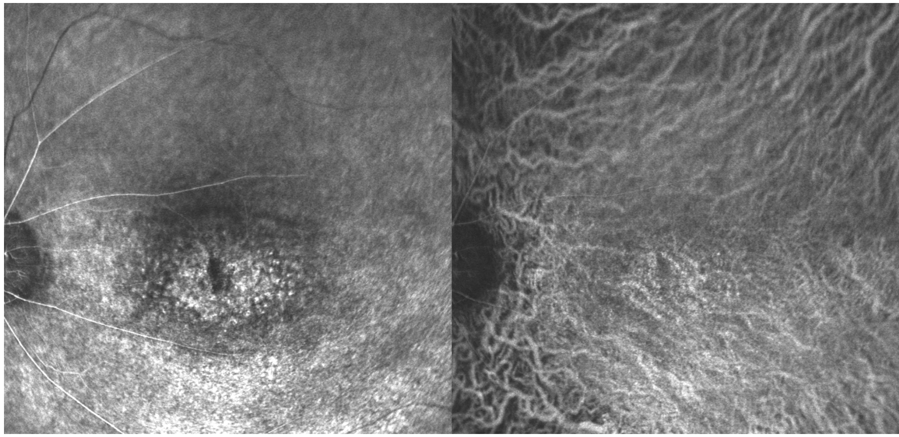

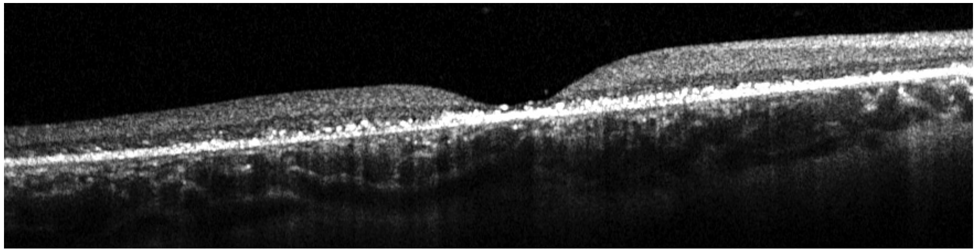

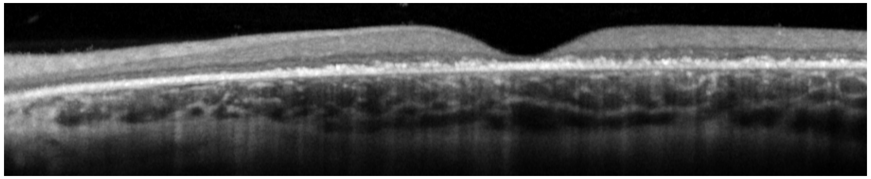

Subject 5 – BDrh-05-CR – Color, FA

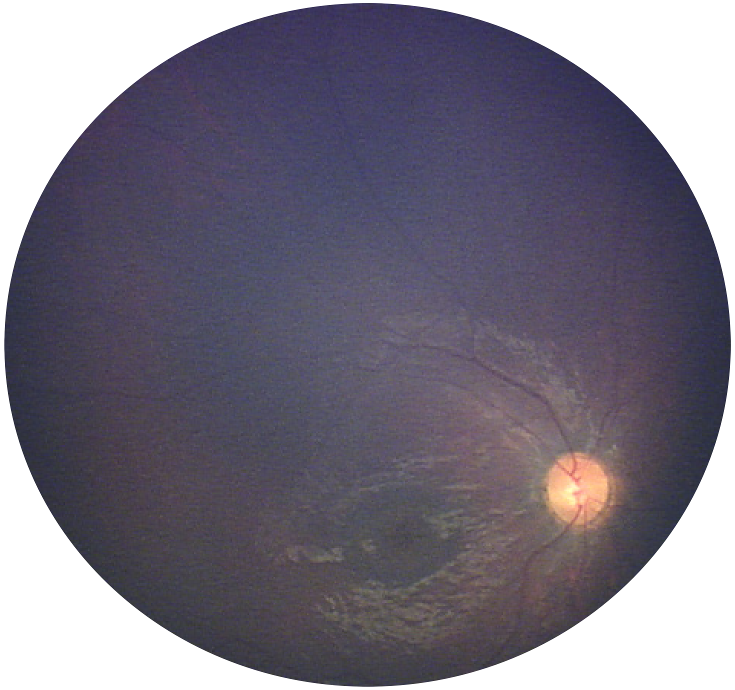

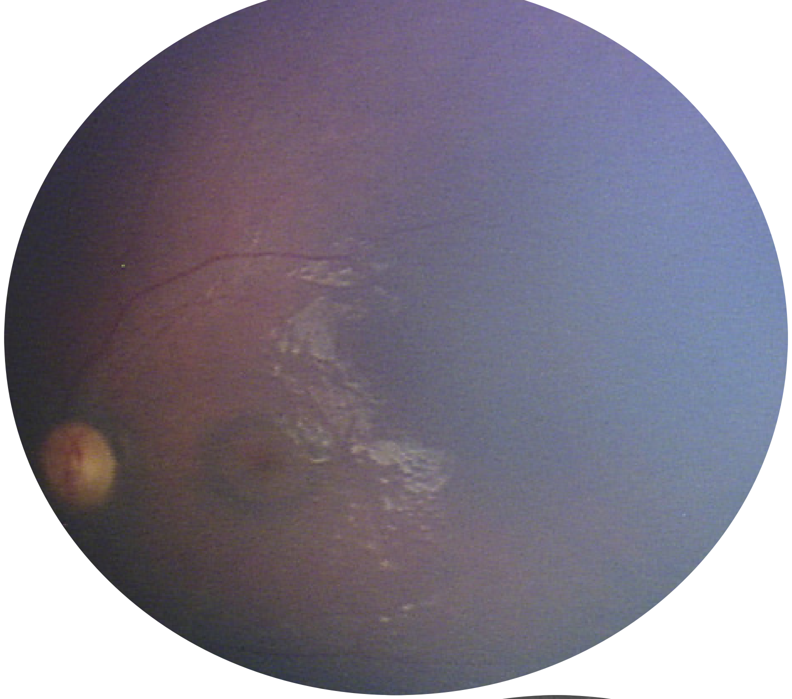

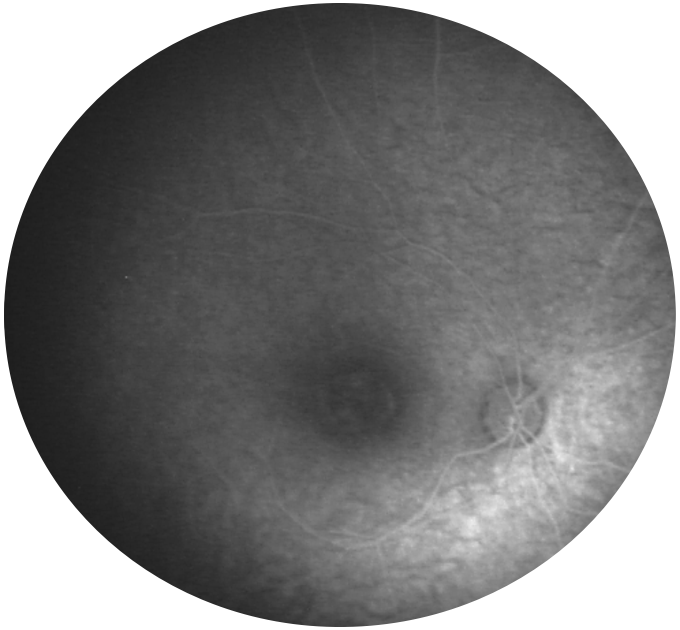

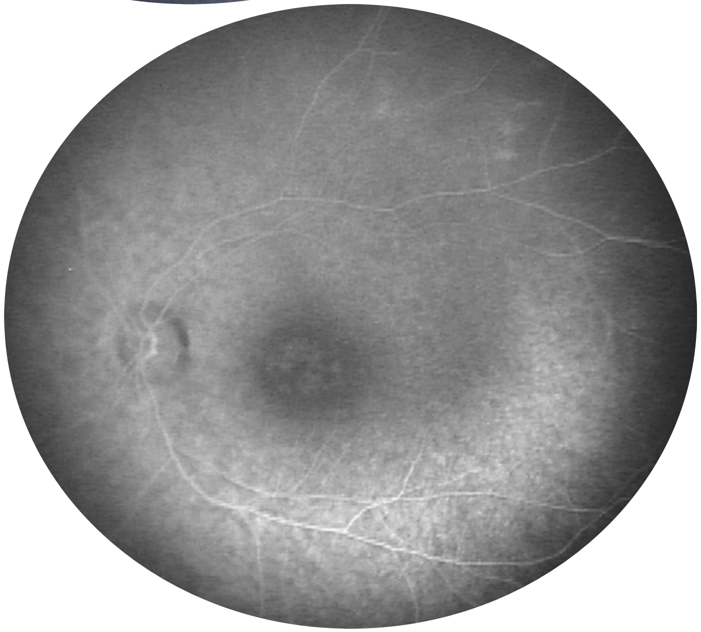

Subject 6 – BDrh-06-CF – Color, FA

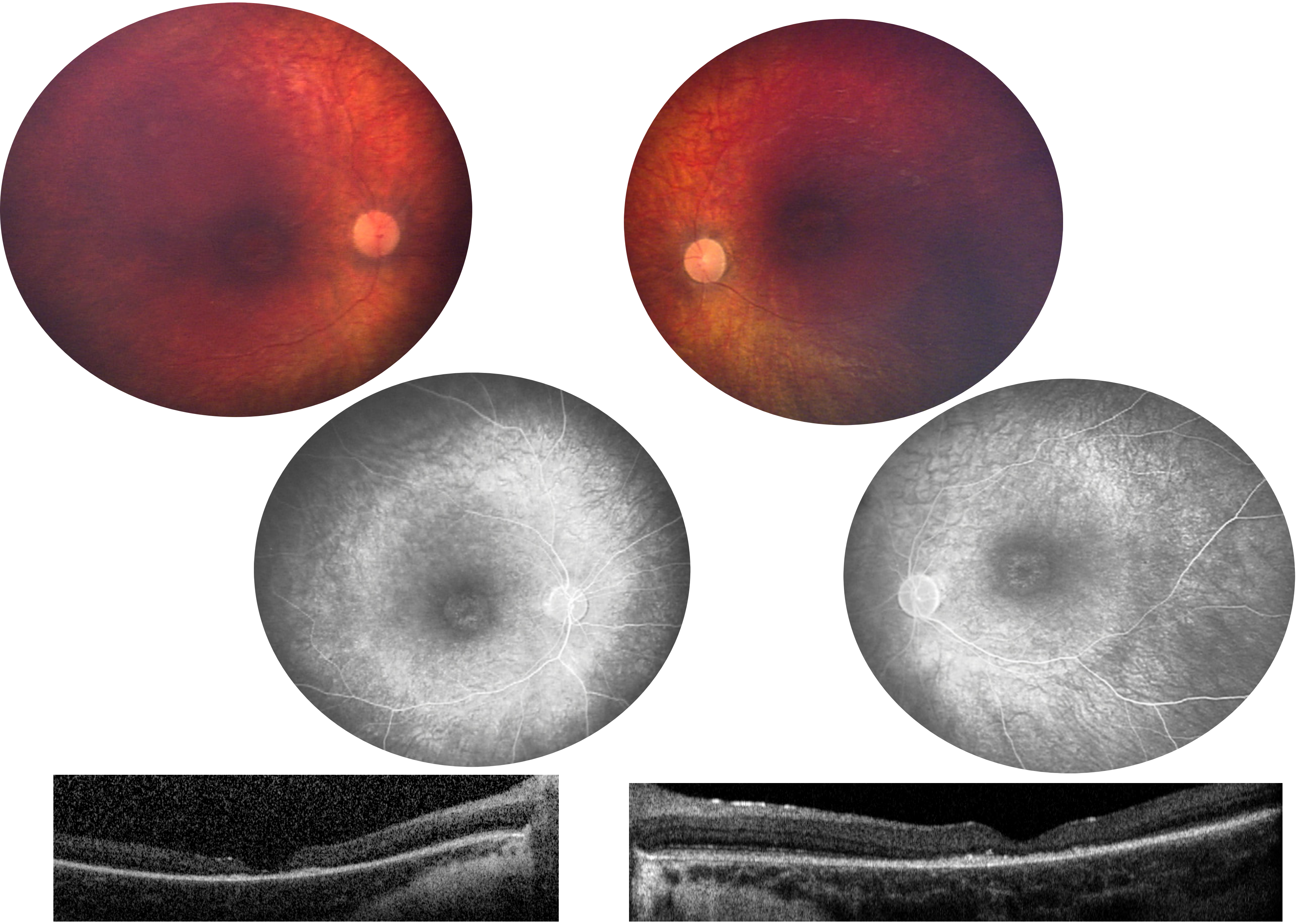

Subject 7 – BDrh-07-OF – Color, FA, ICGA, OCT

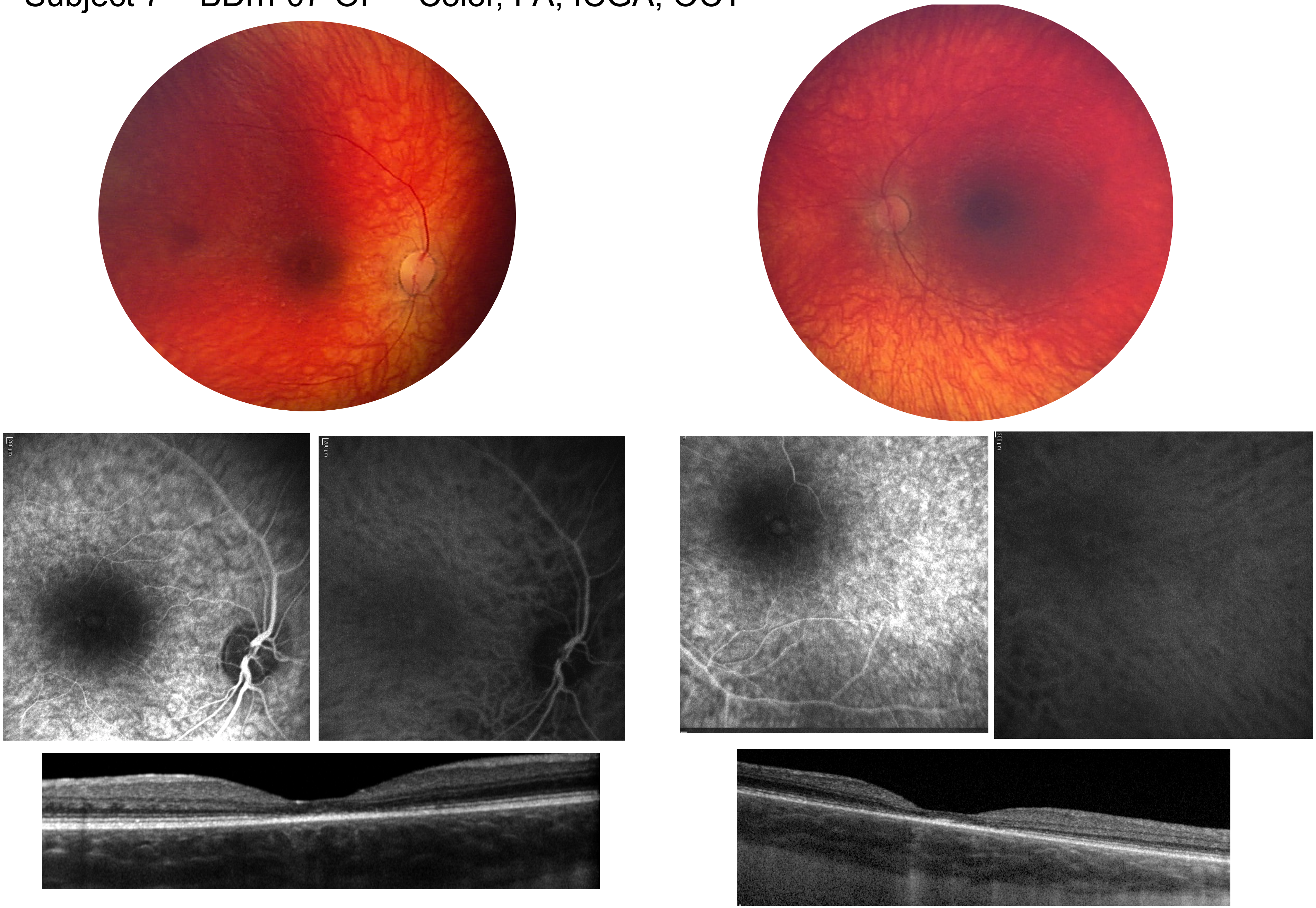

Subject 8 – BDrh-09-FM – Color, FA, ICGA, OCT

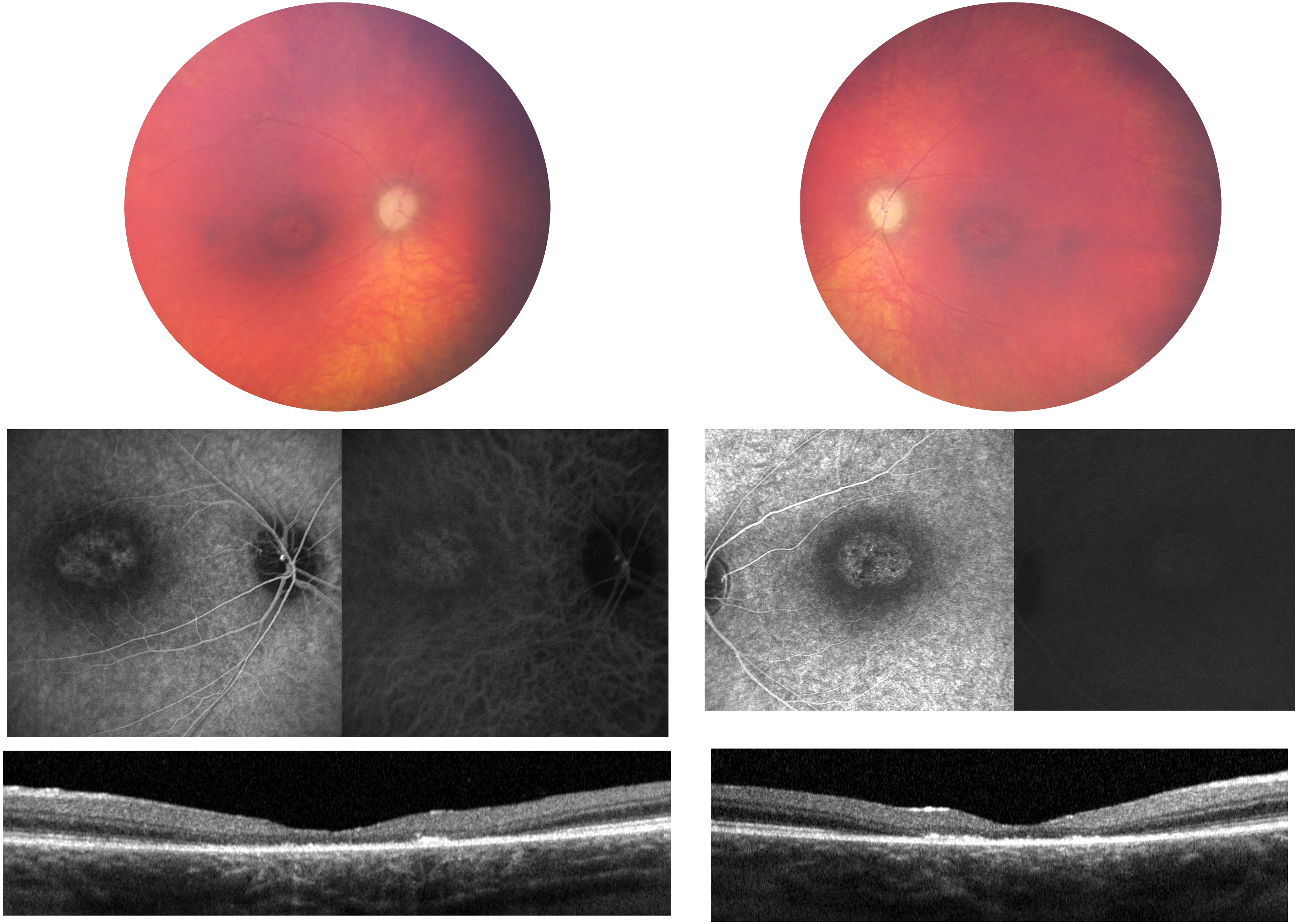

Subject 9 – BDrh-10-SO – Color, FA, ICGA, OCT

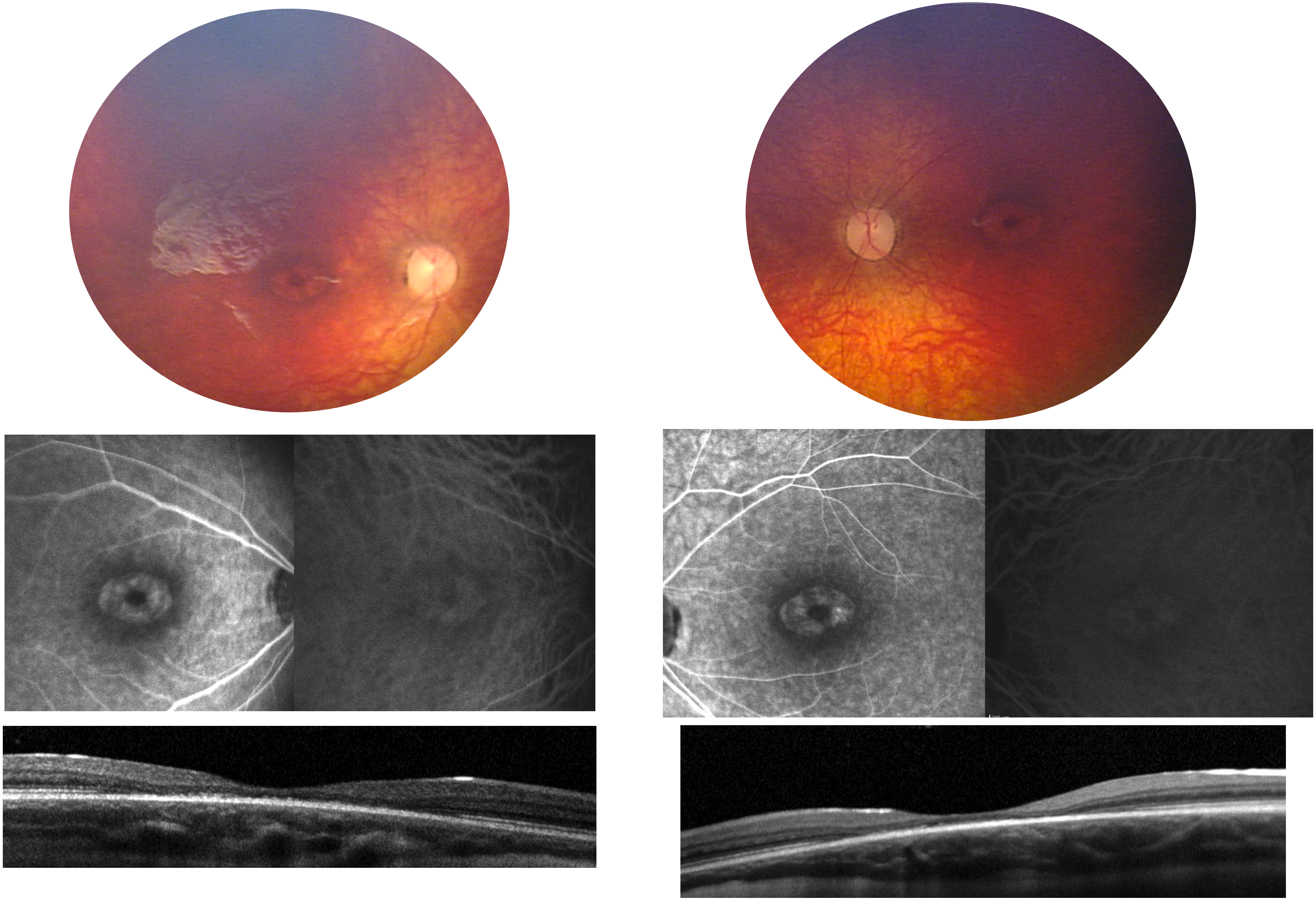

Subject 10 – BDrh-11-UC – Color, FA, ICGA, OCT

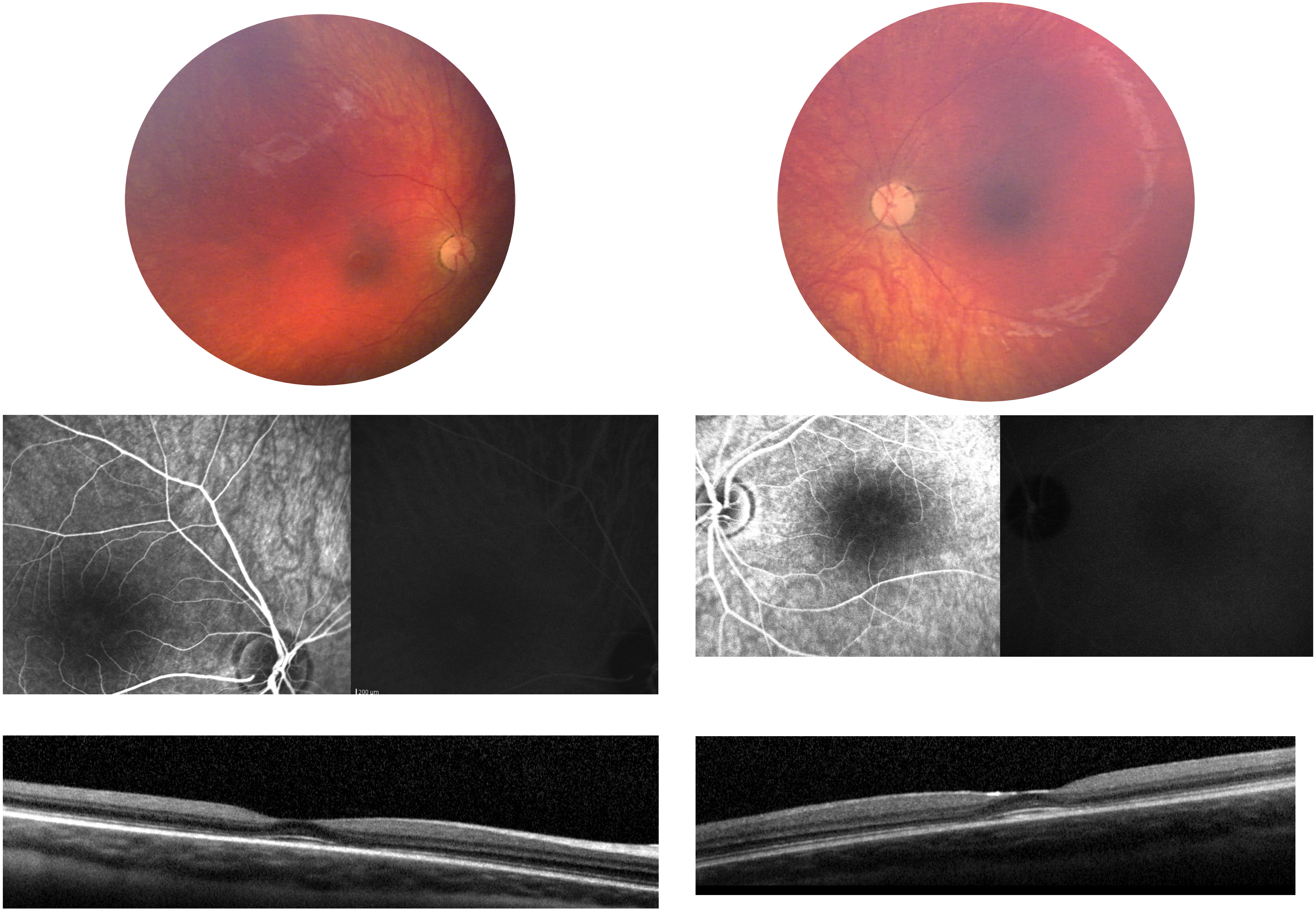

Subject 11 – BDrh-12-FB – Color, FA, ICGA, OCT

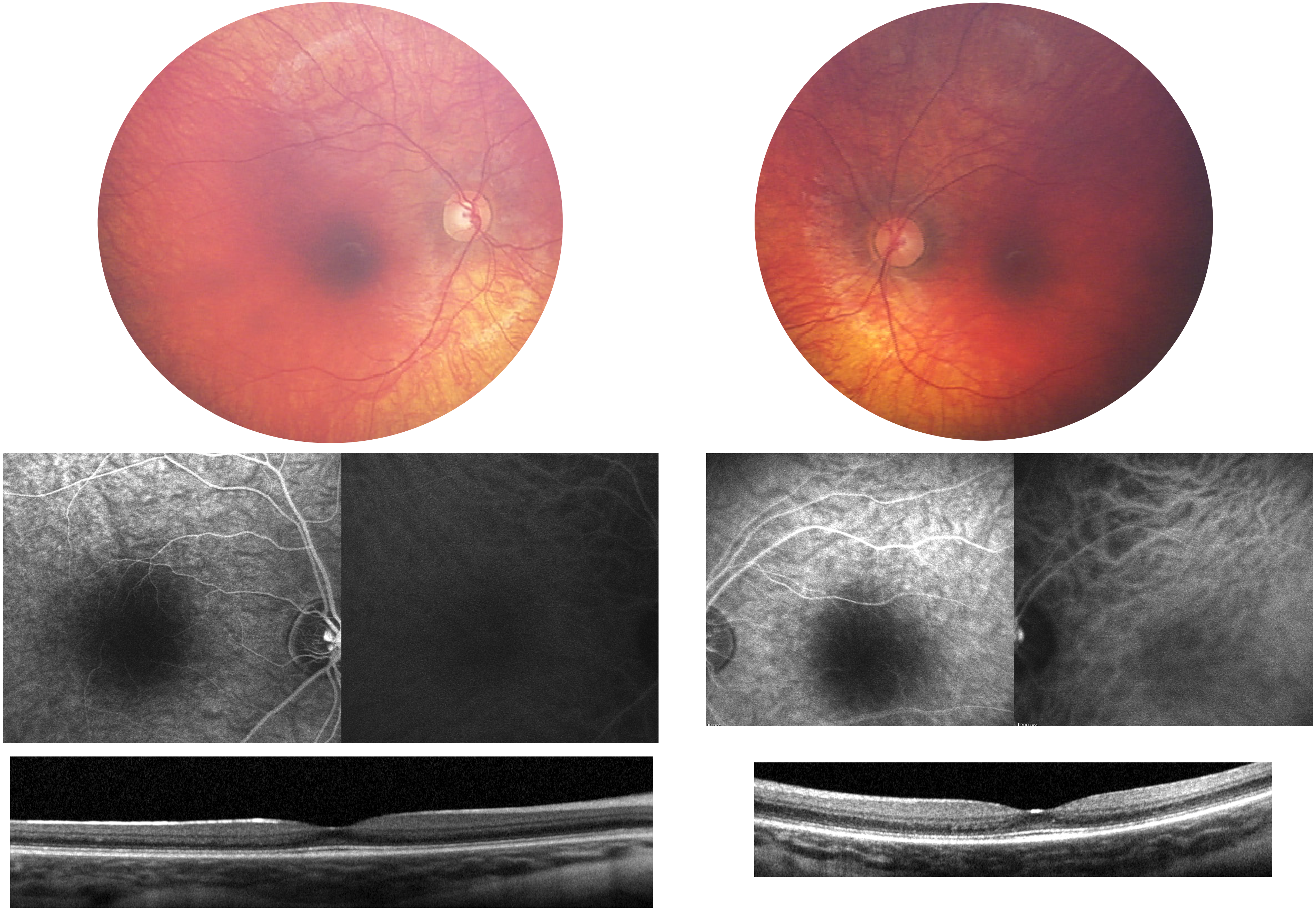

Subject 12 – BDrh-13-UO – Color, FA, ICGA, OCT

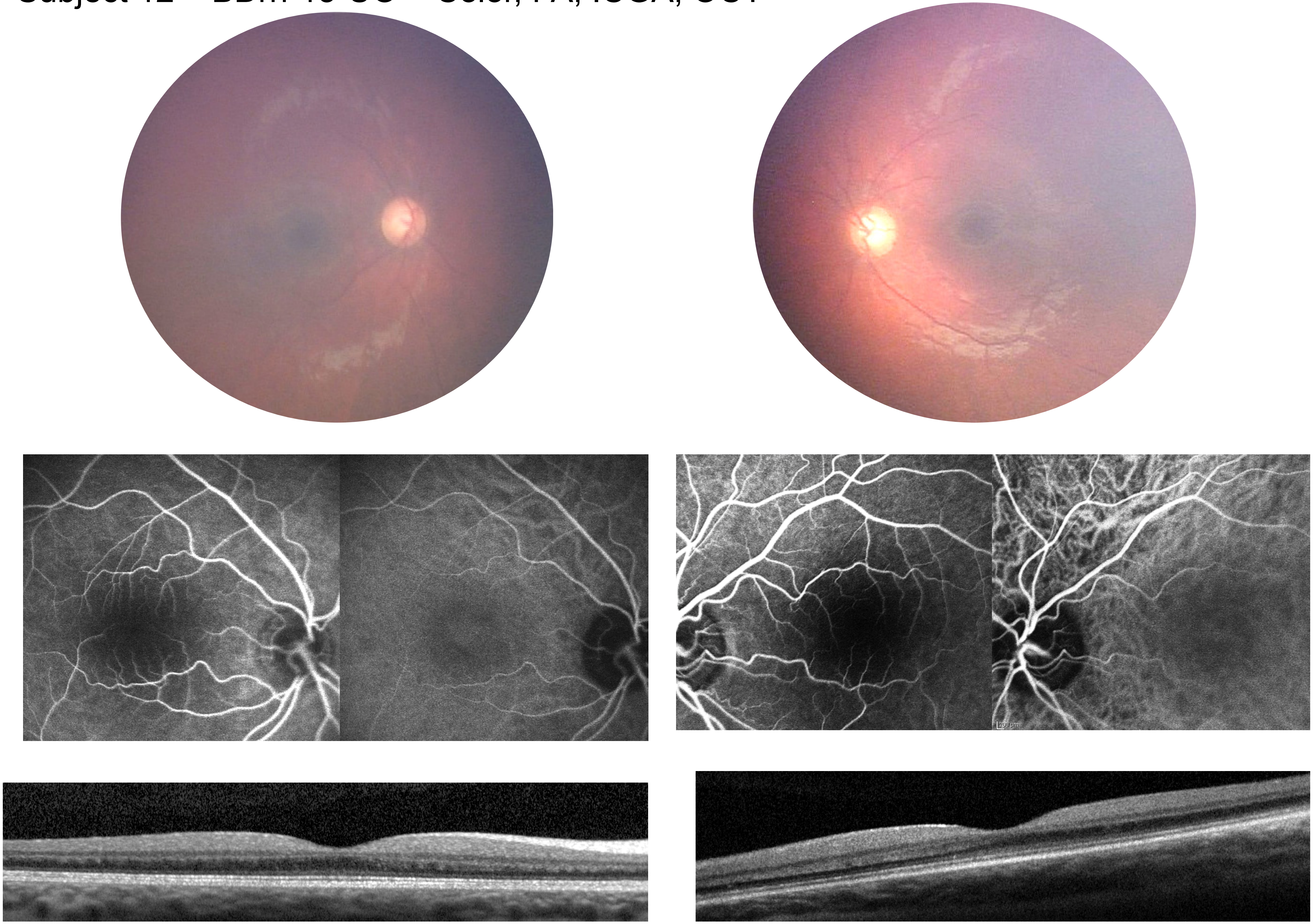

Subject 13 – BDrh-14-UC – Color, FA, ICGA, OCT

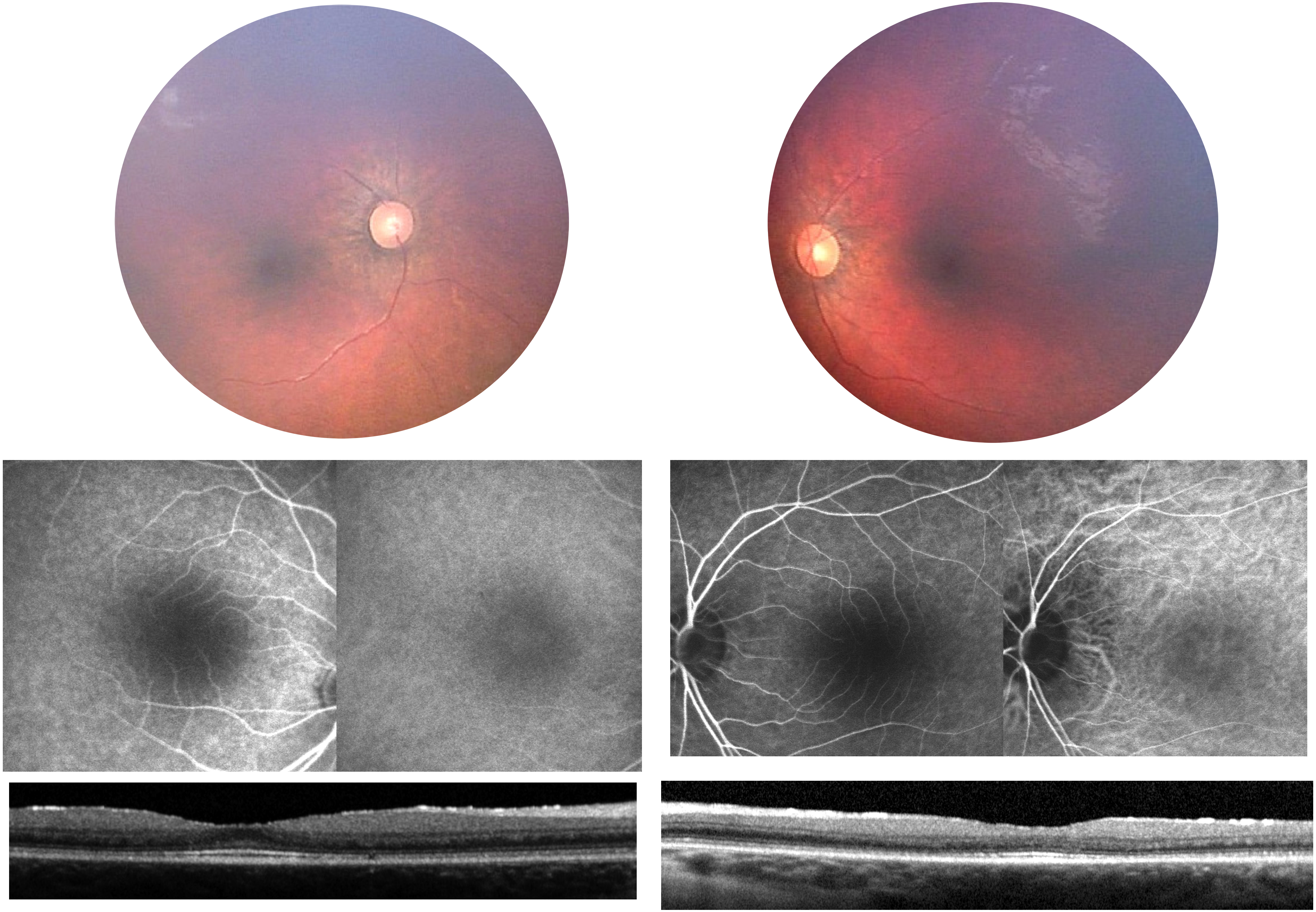

Subject 14 – BDrh-15-BG – Color, FA, ICGA, OCT

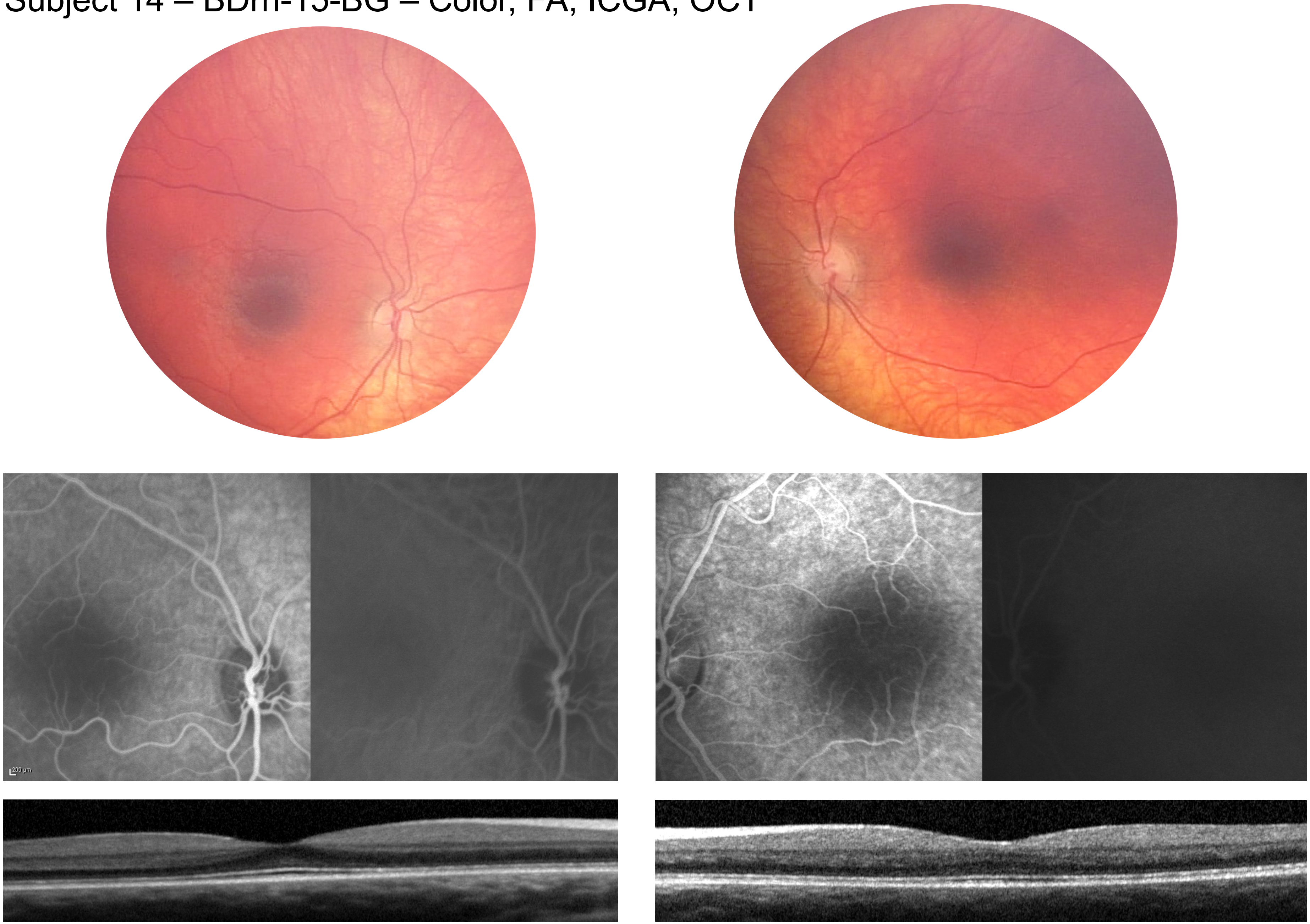

Subject 15 – BDrh-16-PS – Color, FA, ICGA, OCT

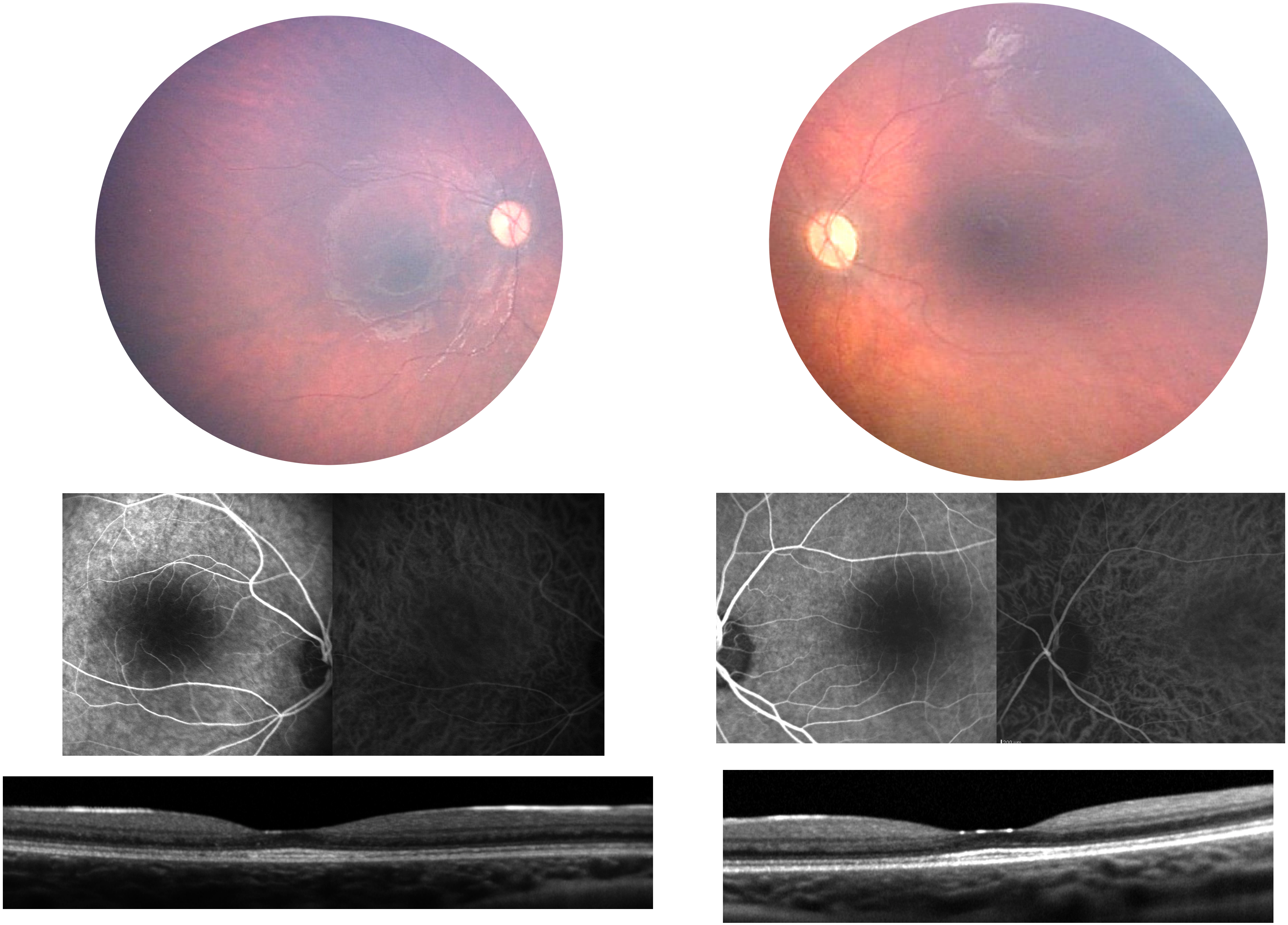

Subject 16 – BDrh-17-TS – Color, FA, ICGA, OCT

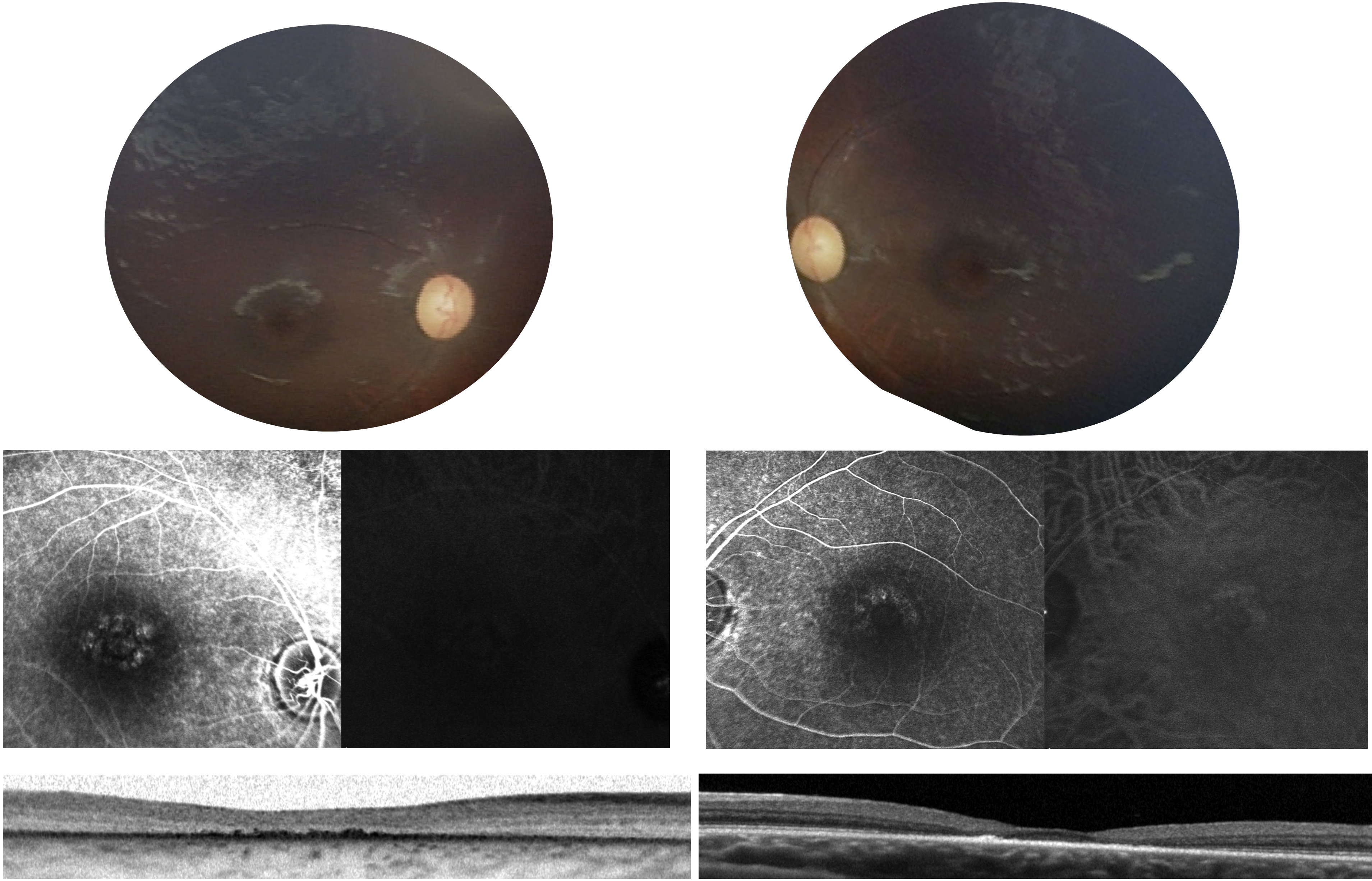

Subject 17 – BDrh-18-FD – Color, FA, ICGA, OCT

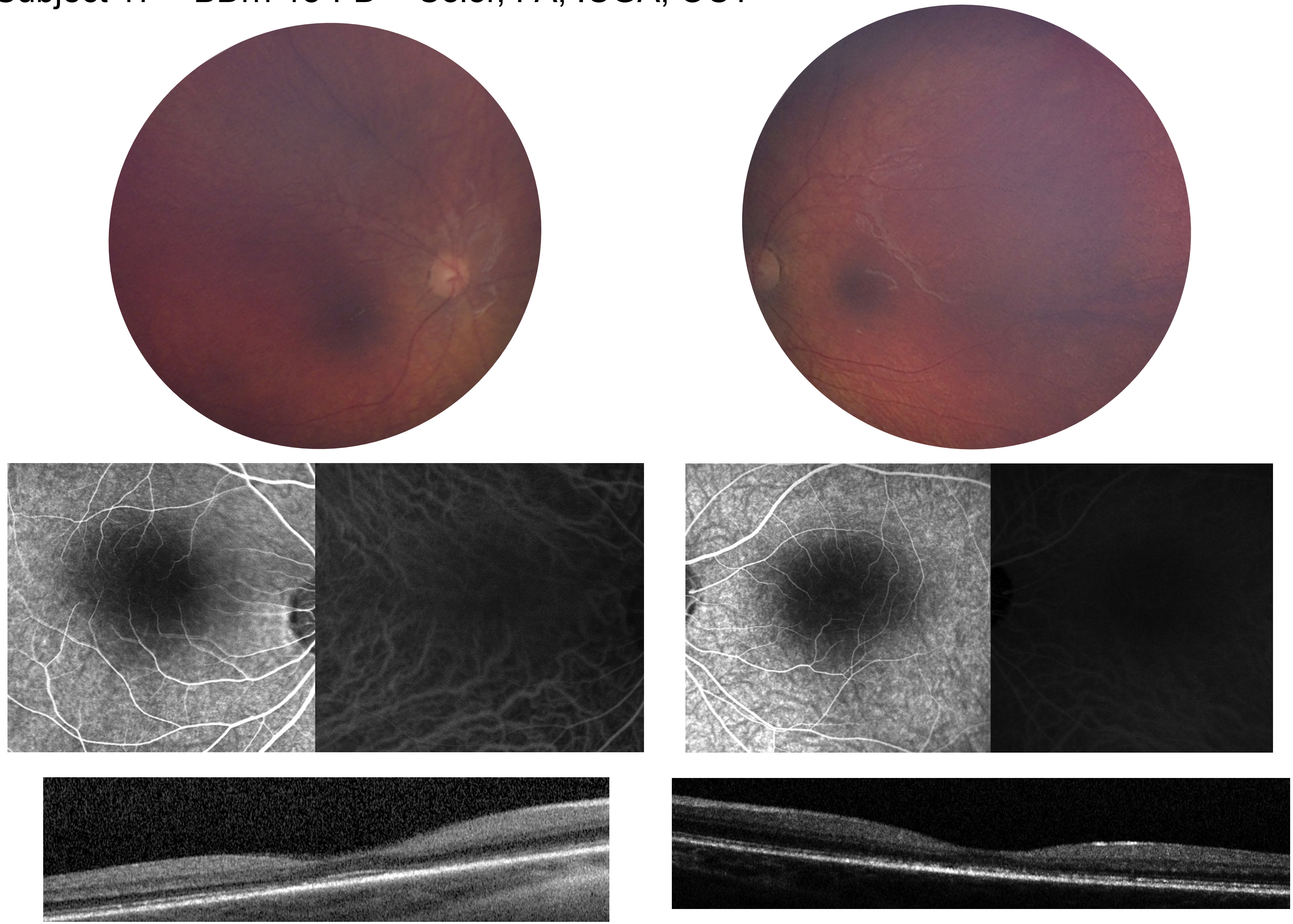

Subject 18 – BDrh-19-UF – Color, FA, ICGA, OCT

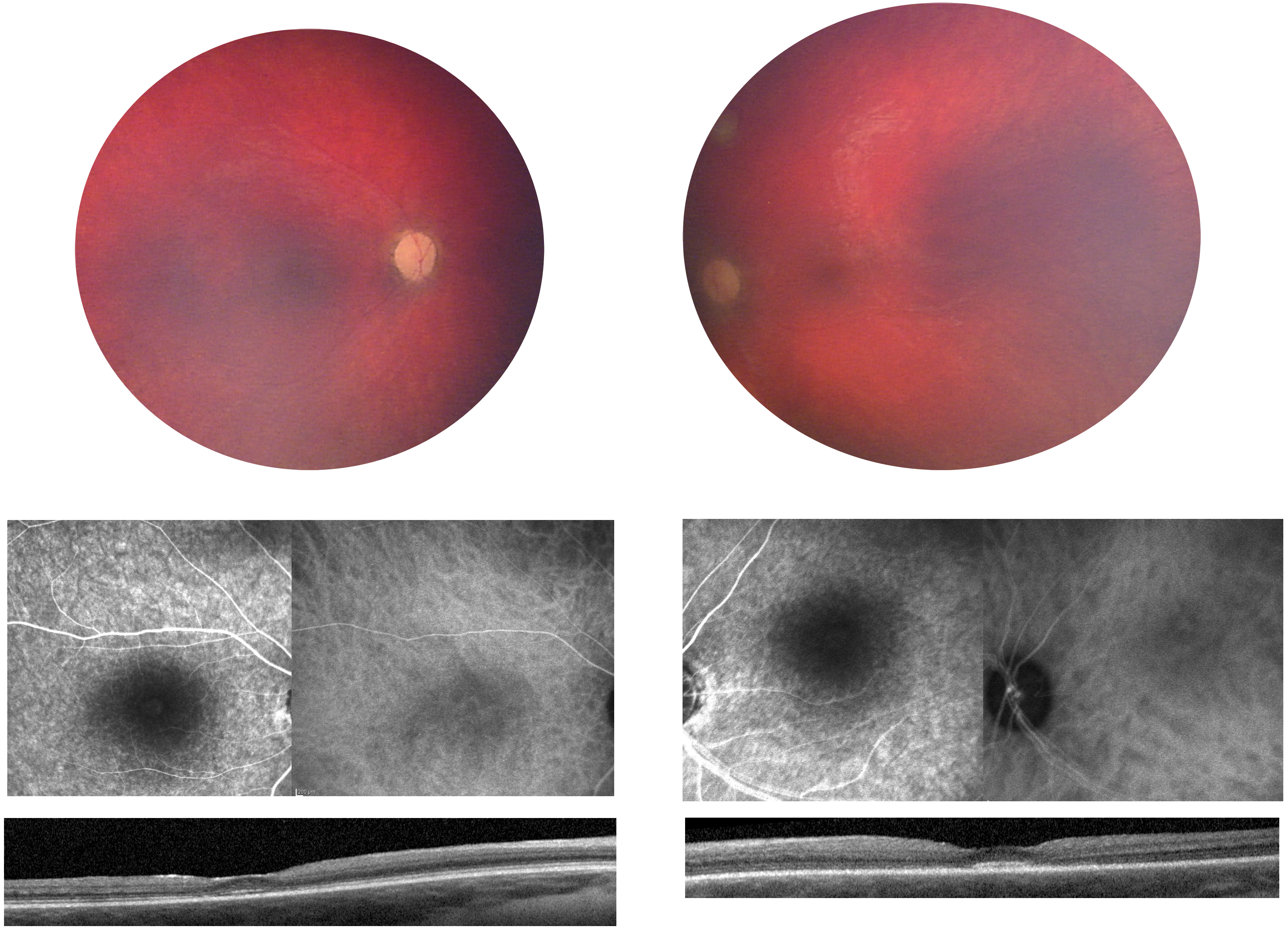

Subject 19 – BDrh-20-SH – Color, FA, ICGA, OCT

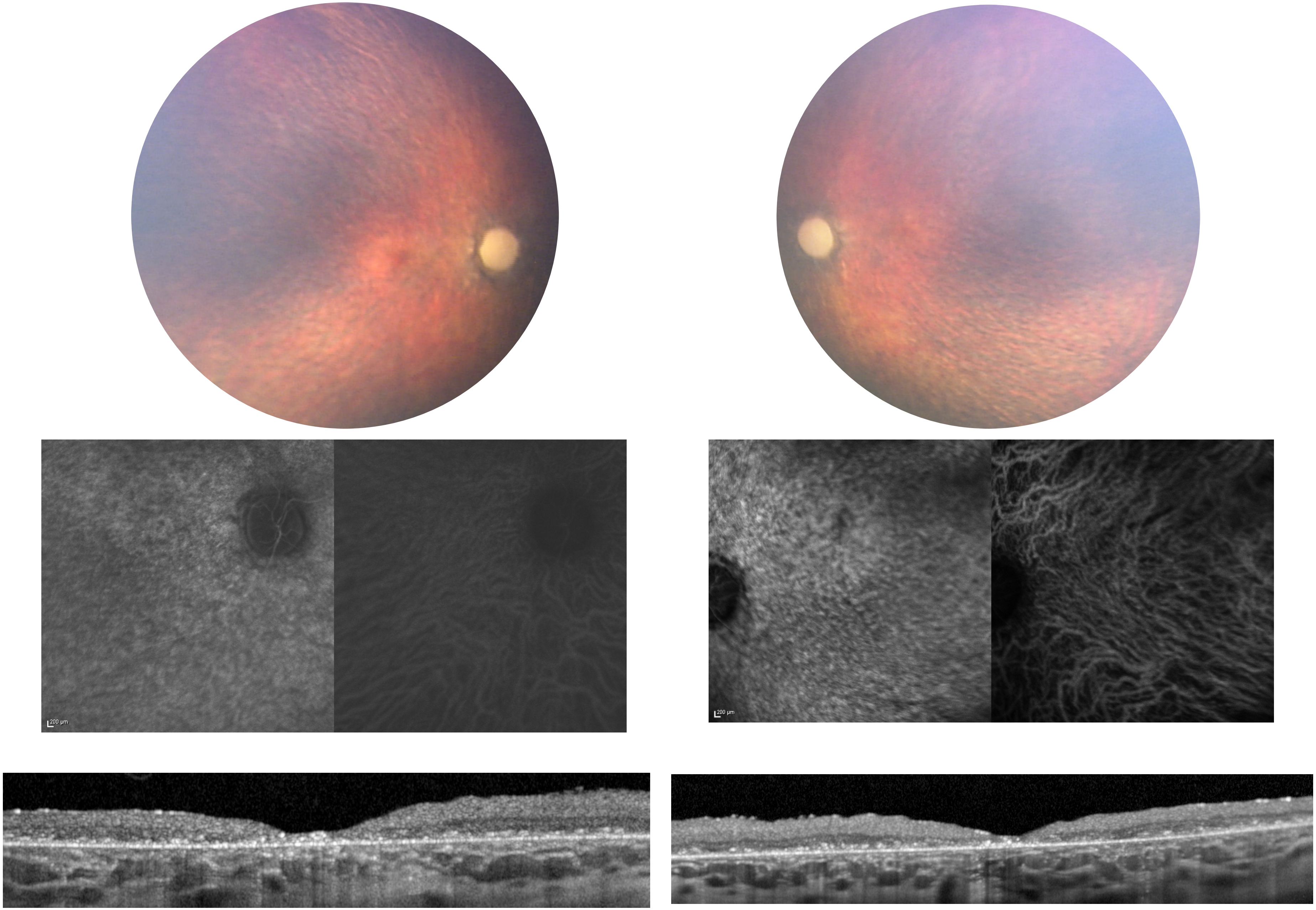

Subject 20 – BDrh-21-JT – Color, FA, ICGA, OCT

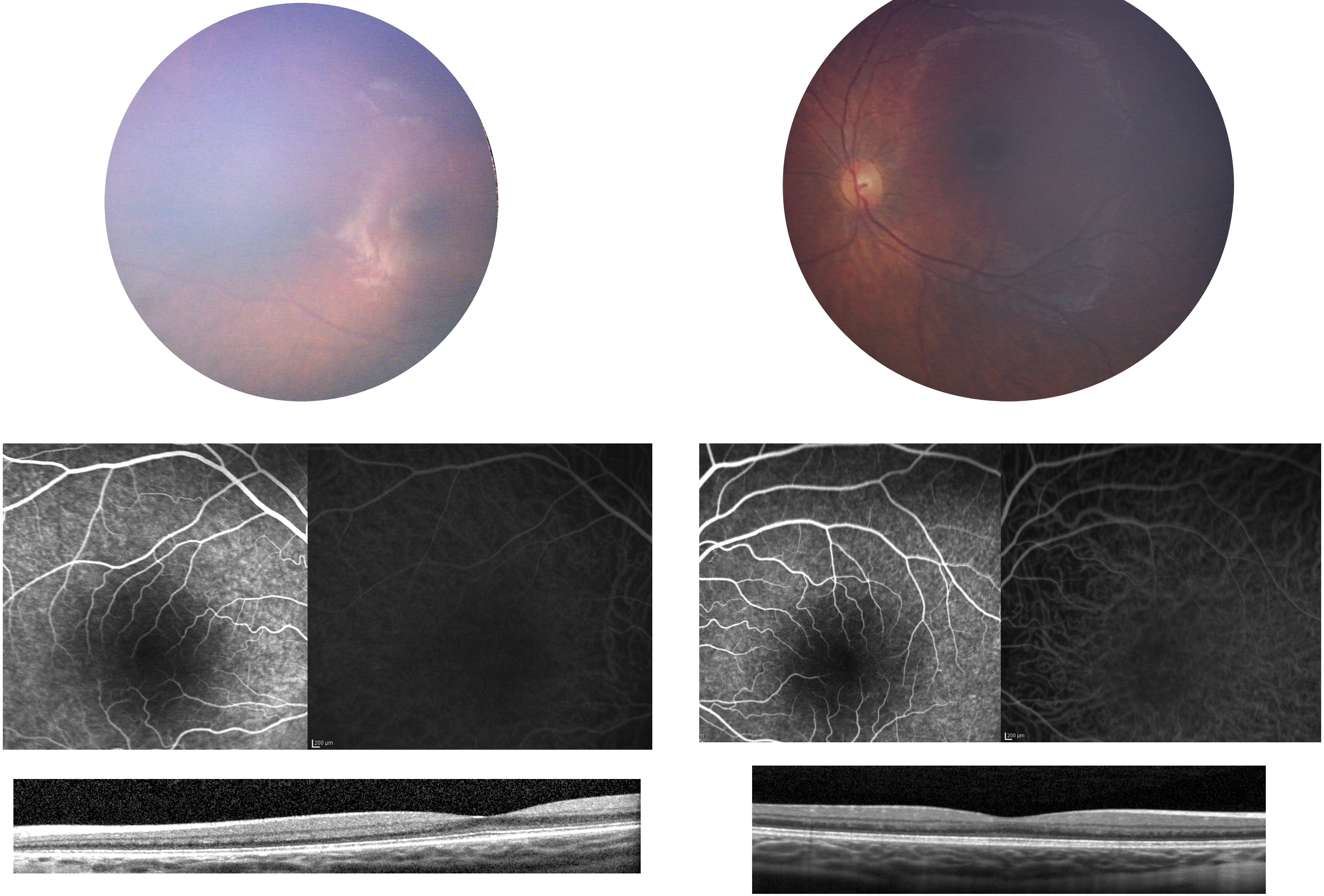

Subject 21 – BDrh-22-LK– Color, FA

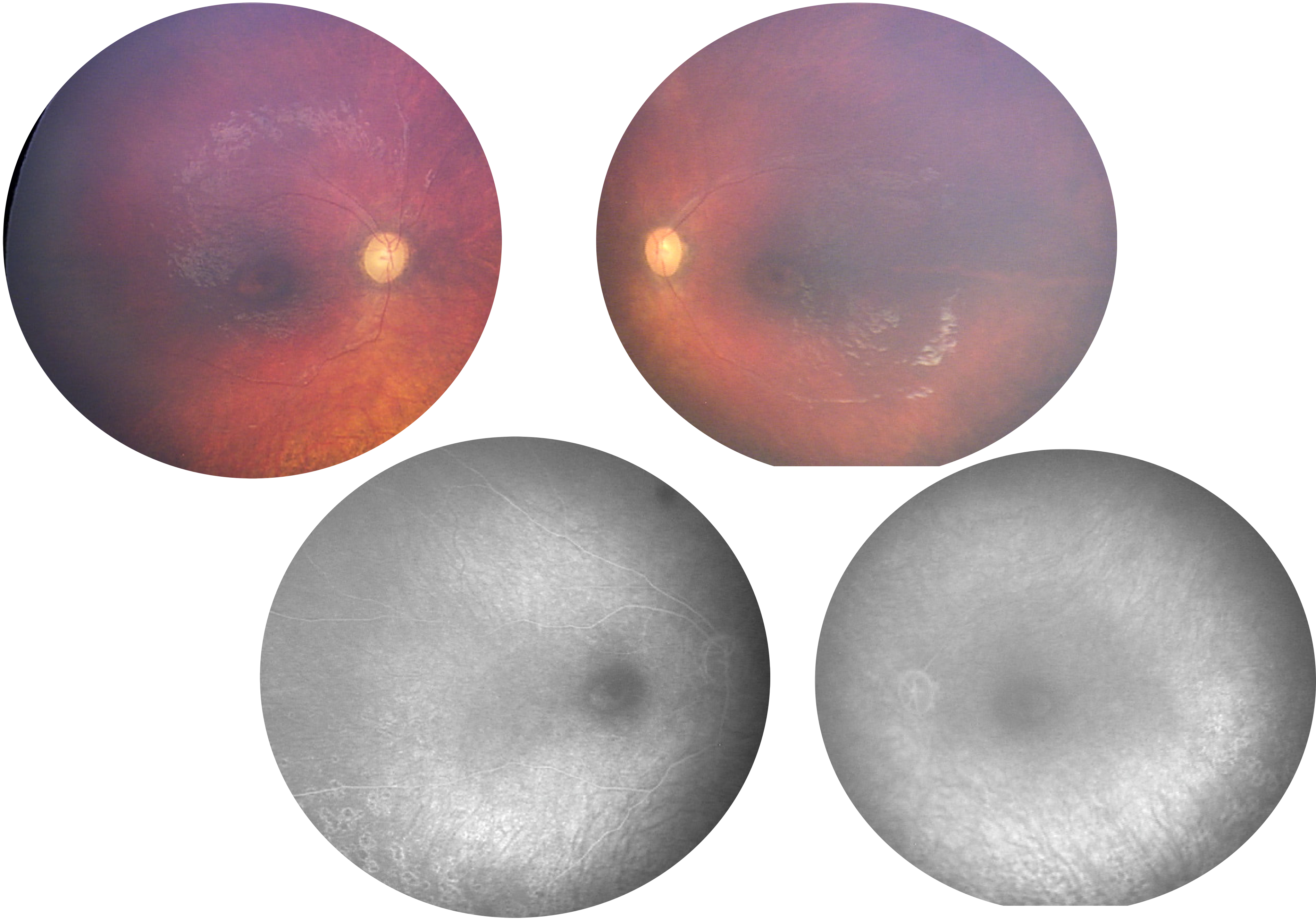

Subject 22 – BDrh-23-SC– Color, FA

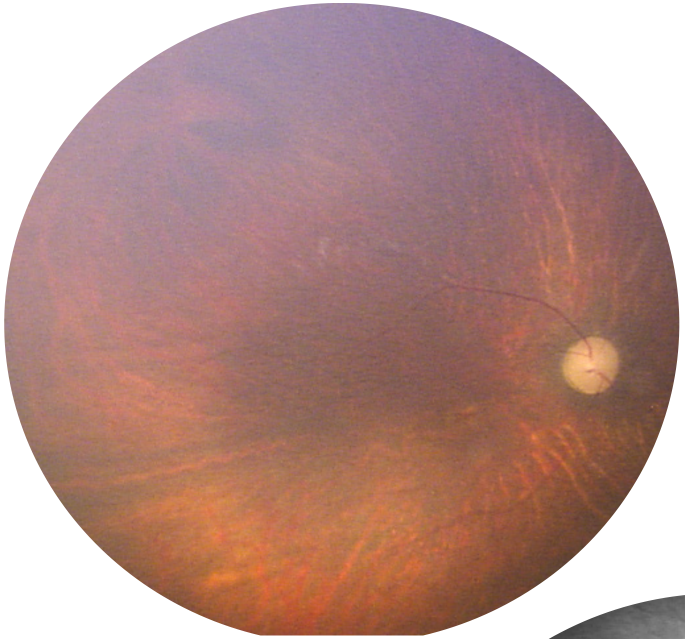

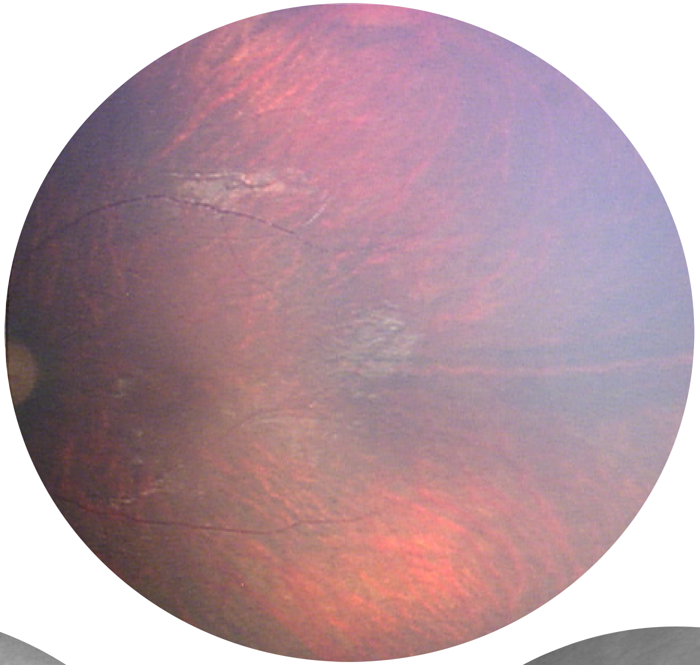

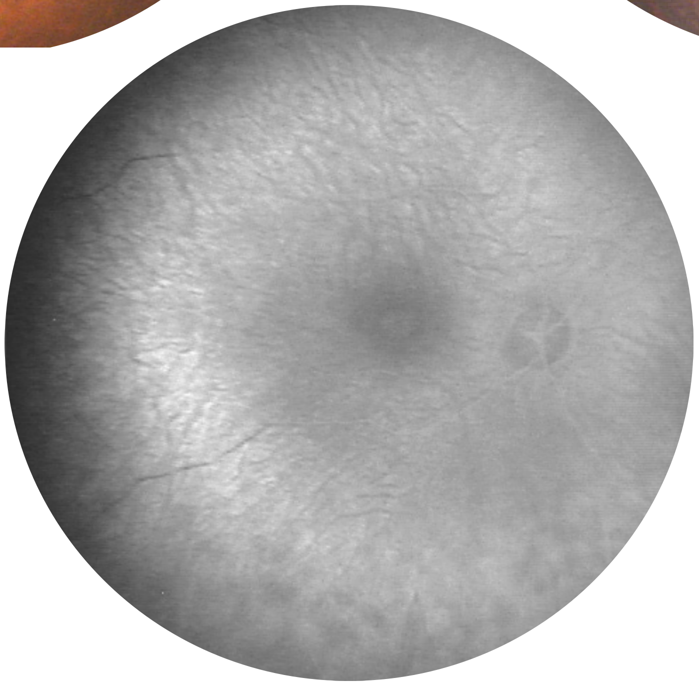

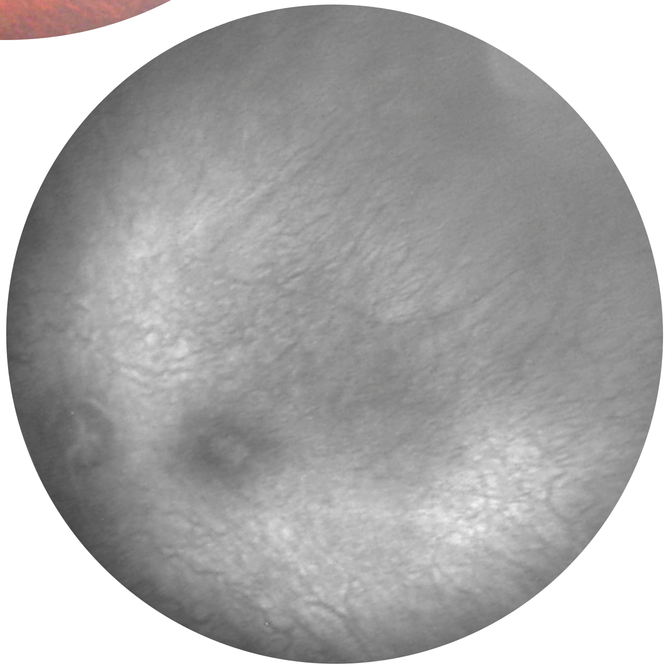

Subject 23 – BDrh-24-BA– Color, FA

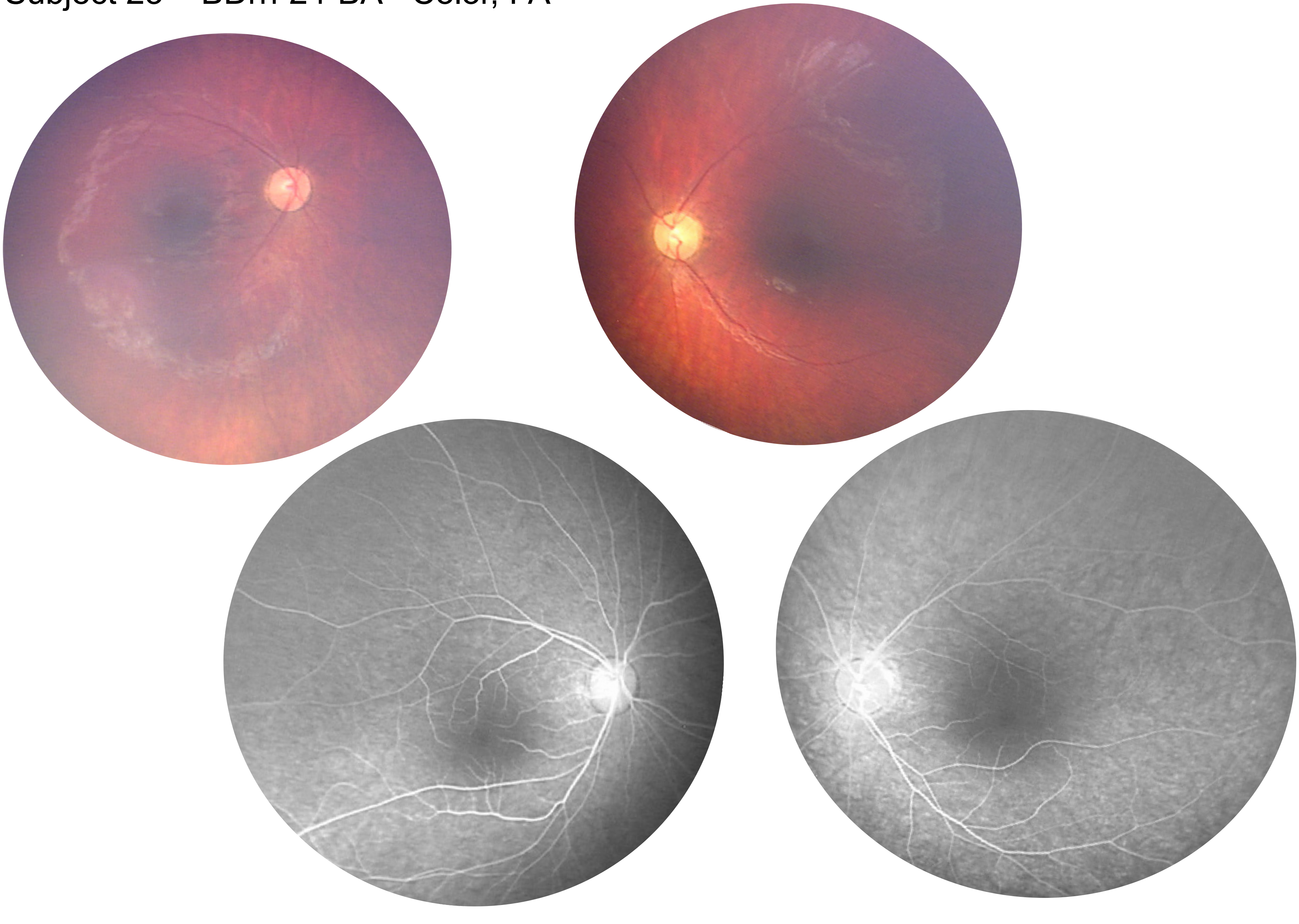

Subject 24 – BDrh-25-MT– Color, FA

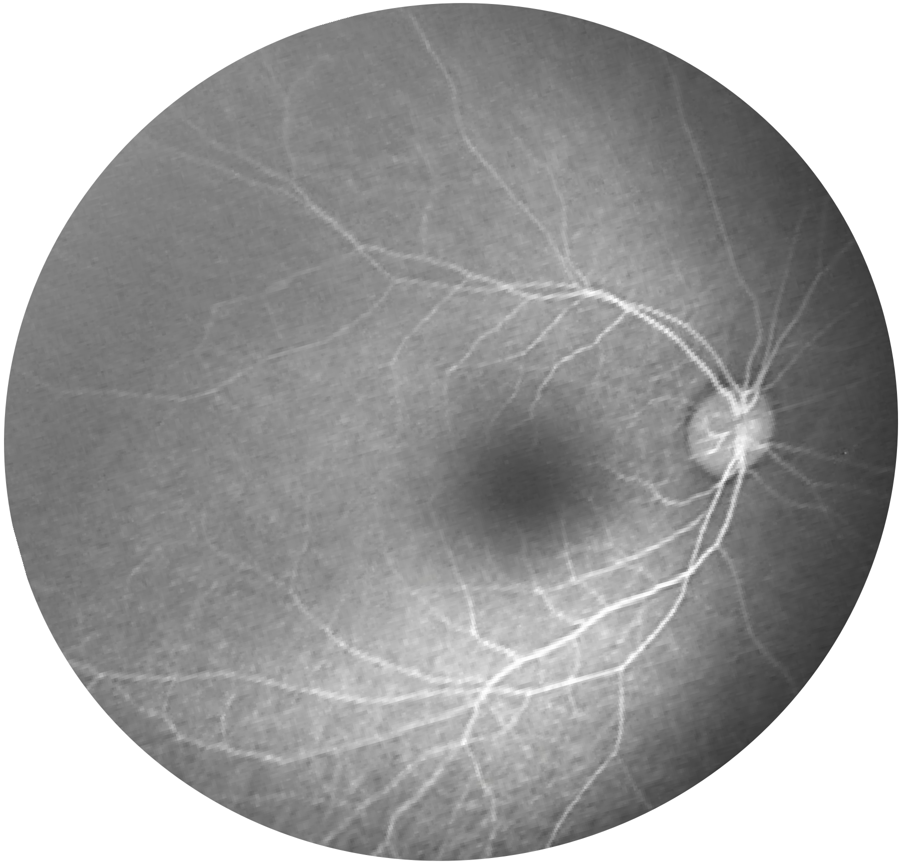

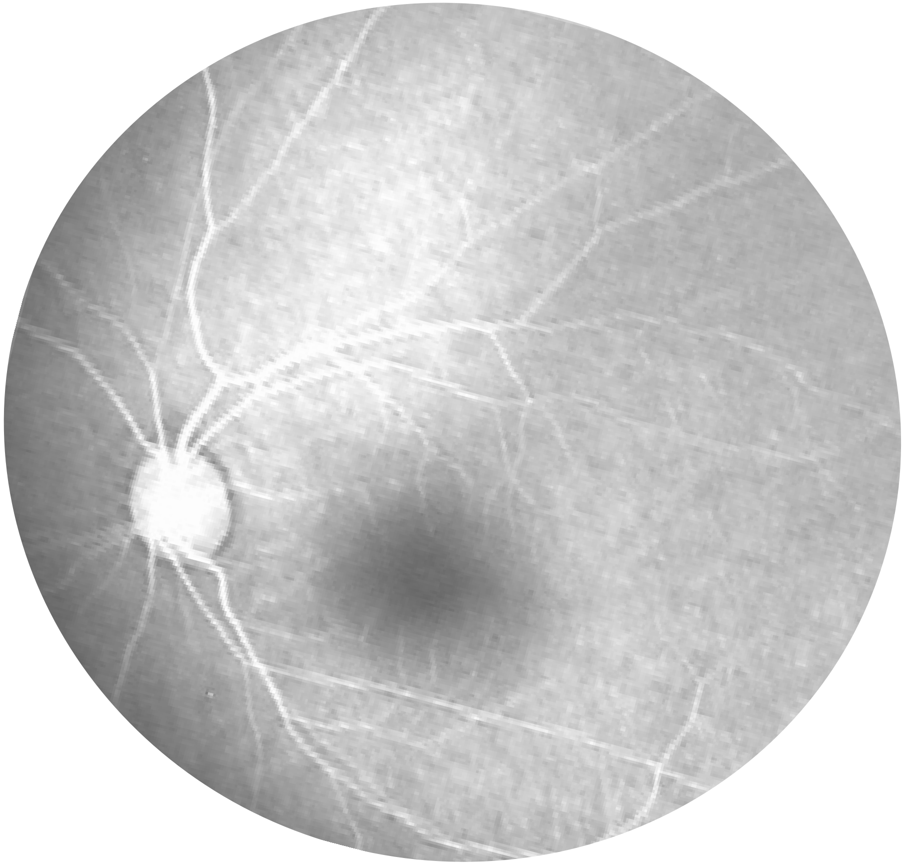

Subject 25 – BDrh-26-NV– Color, FA

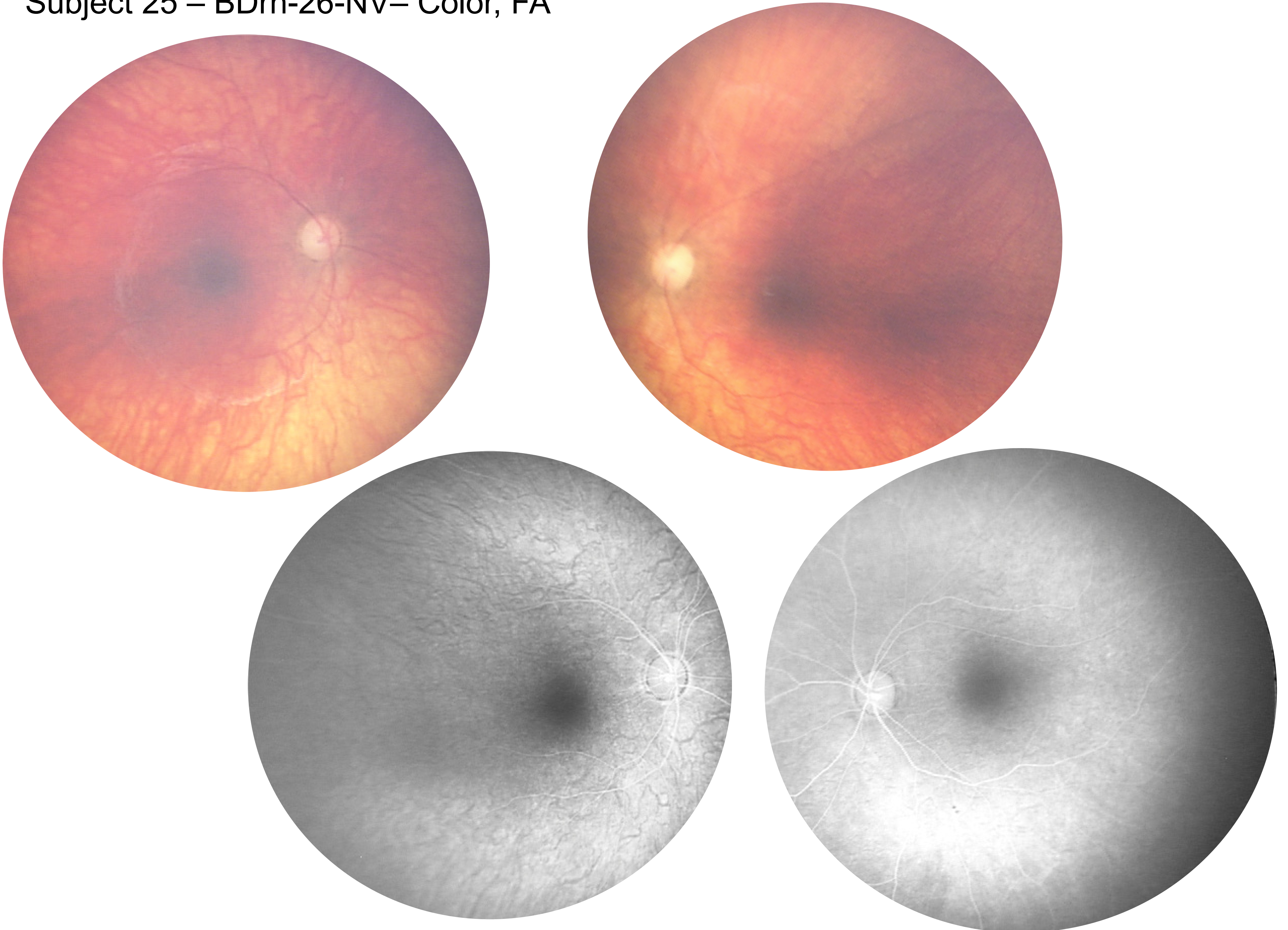

Supplement: Figure S1 — Representative images from the 50 eyes of the 25 patients with LINCL. Patients 1, 2, 5 and 21-25 underwent dilated fundus photography and FA; patients 3 and 6 had dilated fundus photography, FA and SD-OCT; patients 4, and 7-20 underwent the most comprehensive evaluation with dilated color photography, FA, ICGA and SD-OCT. FA – fluorescein angiogram, ICGA – indocyanine green angiogram, SD-OCT – spectral domain optical coherence tomography. (PDF) [file pone.0073128.s001.pdf]
